# Application of data integration for rice bacterial strain selection by combining their osmotic stress response and plant growth-promoting traits

**DOI:** 10.3389/fmicb.2022.1058772

**Published:** 2022-12-15

**Authors:** Arun Kumar Devarajan, Marika Truu, Sabarinathan Kuttalingam Gopalasubramaniam, Gomathy Muthukrishanan, Jaak Truu

**Affiliations:** ^1^Institute of Molecular and Cell Biology, University of Tartu, Tartu, Estonia; ^2^Department of Plant Pathology, Agricultural College and Research Institute, Tamil Nadu Agricultural University, Killikulam, Tuticorin, India; ^3^Department of Soil Science and Agricultural Chemistry, Agricultural College and Research Institute, Tamil Nadu Agricultural University, Killikulam, Tuticorin, India

**Keywords:** osmotic stress, rice phyllosphere, plant growth-promoting bacteria, data integration, strain selection

## Abstract

Agricultural application of plant-beneficial bacteria to improve crop yield and alleviate the stress caused by environmental conditions, pests, and pathogens is gaining popularity. However, before using these bacterial strains in plant experiments, their environmental stress responses and plant health improvement potential should be examined. In this study, we explored the applicability of three unsupervised machine learning-based data integration methods, including principal component analysis (PCA) of concatenated data, multiple co-inertia analysis (MCIA), and multiple kernel learning (MKL), to select osmotic stress-tolerant plant growth-promoting (PGP) bacterial strains isolated from the rice phyllosphere. The studied datasets consisted of direct and indirect PGP activity measurements and osmotic stress responses of eight bacterial strains previously isolated from the phyllosphere of drought-tolerant rice cultivar. The production of phytohormones, such as indole-acetic acid (IAA), gibberellic acid (GA), abscisic acid (ABA), and cytokinin, were used as direct PGP traits, whereas the production of hydrogen cyanide and siderophore and antagonistic activity against the foliar pathogens *Pyricularia oryzae* and *Helminthosporium oryzae* were evaluated as measures of indirect PGP activity. The strains were subjected to a range of osmotic stress levels by adding PEG 6000 (0, 11, 21, and 32.6%) to their growth medium. The results of the osmotic stress response experiments showed that all bacterial strains accumulated endogenous proline and glycine betaine (GB) and exhibited an increase in growth, when osmotic stress levels were increased to a specific degree, while the production of IAA and GA considerably decreased. The three applied data integration methods did not provide a similar grouping of the strains. Especially deviant was the ordination of microbial strains based on the PCA of concatenated data. However, all three data integration methods indicated that the strains *Bacillus altitudinis* PB46 and *B*. *megaterium* PB50 shared high similarity in PGP traits and osmotic stress response. Overall, our results indicate that data integration methods complement the single-table data analysis approach and improve the selection process for PGP microbial strains.

## Introduction

Global food production has been significantly affected by climate change and the evolution of pests and pathogens (FAO, 2022). Rice is one of the world’s most important food crops, but it is highly vulnerable to numerous abiotic and biotic stresses (Sandhu et al., 2020). Plants have several mechanisms to adapt to and establish tolerance and resistance to stress (Vats, 2018; Chauhan et al., 2022; Hidangmayum et al., 2022; Sachdev and Ansari, 2022). One such mechanism is symbiosis, wherein beneficial microorganisms living in or on plant organs directly and/or indirectly support plant growth and protect them from biotic and abiotic stresses (Shinwari et al., 2019). Given this beneficial effect, more attention has been paid to rice microbiome to increase crop yield and achieve sustainable agricultural goals. The microbial community composition of the rice rhizosphere has been extensively investigated using both culture-dependent and -independent approaches (Ding et al., 2019). Although the role of rhizobacteria in crop health improvement under abiotic stress has been widely reported (Ayuso-Calles et al., 2021; Saxena et al., 2021), the contribution of rice phyllosphere and spermosphere microbial communities and populations to this process is only partially understood (Kim and Lee, 2020).

The phyllosphere refers to a plant’s total aboveground surface, representing an unstable habitat, where microbes are subjected to extreme and highly variable environmental factors, such as light intensity, ultraviolet (UV) radiation, temperature, and dryness. Bacteria dominate this habitat, and their number estimated either by cultivation or direct microscopy can be up to 10^7^ cells per cm^2^ on plant leaf surfaces (Vorholt, 2012; Remus-Emsermann and Schlechter, 2018). Microbial genetic and metabolic competence has been shown to help bacteria overcome and survive extreme environments (Sessitsch et al., 2012; Shu and Huang, 2022). Venkatachalam et al. (2016) demonstrated that the adaptability and functionality of culturable rice microbial communities were related to their diversity and abundance in the phyllosphere, while their ability to stimulate plant growth was strongly influenced by the rice cultivation method. *Bacillus* species can be highly resistant to extreme abiotic stress factors, such as UV radiation, high and low temperatures, and dryness, by forming resistant endospores and having higher survival rates than other bacterial species in the phyllosphere (Setlow, 2014; Saleem et al., 2017).

Under high temperatures and desiccation stress, microorganisms regularly encounter osmotic stress in the phyllosphere habitat. Under osmotic stress, most bacteria produce compatible osmolytes, such as proline, glycine betaine (GB), ectoine, trehalose, and sucrose (Santos and Da Costa, 2002). Proline reduces osmotic stress by acting as a chemical chaperone that directly breaks down the reactive oxygen species produced during stress and by means of an indirect mechanism that activates the signaling pathways that promote cell survival (Liang et al., 2013). In contrast, quaternary ammonium compounds, such as GB and choline, serve as osmoprotectants. Choline is a precursor of GB, and during osmotic stress, a certain level of choline is oxidized to GB by choline oxidase and betaine aldehyde dehydrogenase (Fitzsimmons et al., 2012). It has been demonstrated that plant growth-promoting bacteria (PGPB) that accumulate osmolytes reduce plant salinity stress (Upadhyay et al., 2011, 2012). Therefore, to understand the osmotic stress tolerance of microorganisms, it is essential to investigate their osmolyte production potential.

The levels of phytohormones in plants significantly affect their growth by regulating metabolism and defense mechanisms (Egamberdieva et al., 2017). Many plant-associated bacteria can produce beneficial phytohormones, mainly indole-acetic acid (IAA), gibberellic acid (GA), cytokinin, and abscisic acid (ABA; Belimov et al., 2014; Nutaratat et al., 2017; Baliyan et al., 2022; Mekureyaw et al., 2022). Some microbes support host plants indirectly by preventing the growth and infestation of pests and pathogens. Such microbes produce a variety of compounds that inhibit the growth of competing organisms. Some of the most important antagonistic mechanisms involve the production of hydrogen cyanide (HCN), which affects cellular respiration and siderophore production, leads to iron chelation, and limits iron accessibility to pathogens (Sayyed et al., 2013; Muthukumar et al., 2022; Singh et al., 2022). Such behavior of beneficial bacteria against host pathogens can suppress diseases in *in vitro* and *in planta* (Ramakrishna et al., 2019; Mahmud et al., 2021). Therefore, the application of phyllosphere bacteria that can produce phytohormones, have antagonistic activity against pathogens, and tolerate osmotic stress can be greatly beneficial in improving rice health through the alleviation of biotic and abiotic stress.

To select the most promising strains for plant applications, the phenotypic and, less often, the genomic data related to PGP characteristics of microbial strains are explored by applying statistical and exploratory analyses. The data obtained for microbial strains are most often examined using univariate statistical methods including *t*-tests, ANOVA, and linear models. Less frequently, univariate data analysis methods are complemented with multivariate approaches, such as cluster analysis or principal component analysis, which allow the elucidation of patterns among strains and relationships with their properties (da Costa et al., 2014). When several types of datasets, such as PGP traits and abiotic and biotic stress response parameters, are generated to characterize the potential of PGP-microbial strains for improving plant growth and health, an integration of these datasets is needed to facilitate the simultaneous identification of important phenotypic and genomic features during strain selection.

If the properties of microbial strains are assessed using different measurement methods and experimental conditions, the data produced by a particular method can be considered single-view data. It is possible to fuse microbial strain data from different perspectives using multi-view learning (MVL), which utilizes the consensual and complementary information between different views of the same set of microbial strains. MVL, also known as data fusion or integration from multiple feature sets, is an emerging direction in multi-view machine learning that can be used to improve generalization performance (Zhao et al., 2017; Li et al., 2018). Picard et al. (2021) recently delineated data integration methods into five different integration strategies (early, mixed, intermediate, late, and hierarchical). Data integration can be applied in two ways, depending on the nature of datasets: horizontal integration, which studies the same parameters across different microbial strains, and vertical integration, which examines multiple sets of variables on the same set of strains. Generally, machine learning methods are highly effective in data integration when datasets are appropriately transformed and combined (Picard et al., 2021; Cai et al., 2022).

Thus far, data integration methods have been applied to multi-omics datasets, where the combination of different layers of molecular information obtained for plant-related microbial communities has been analyzed (Kaul et al., 2016). The suitability of this data analysis approach for characterizing and selecting PGP microbial strains using various types of datasets has not yet been explored. We hypothesized that the application of data integration methods can enhance the selection of PGP microbial strains for plant application if multiple PGP-related datasets are measured for these strains.

The main aim of this study was to test the applicability of three unsupervised machine learning-based data integration methods for simultaneous grouping and trait evaluation of osmotic stress-tolerant PGP bacterial strains isolated from the rice phyllosphere. In addition, a set of univariate and multivariate data analysis methods conventionally used for this purpose was implemented.

## Materials and methods

### Study design

**Figure 1:**
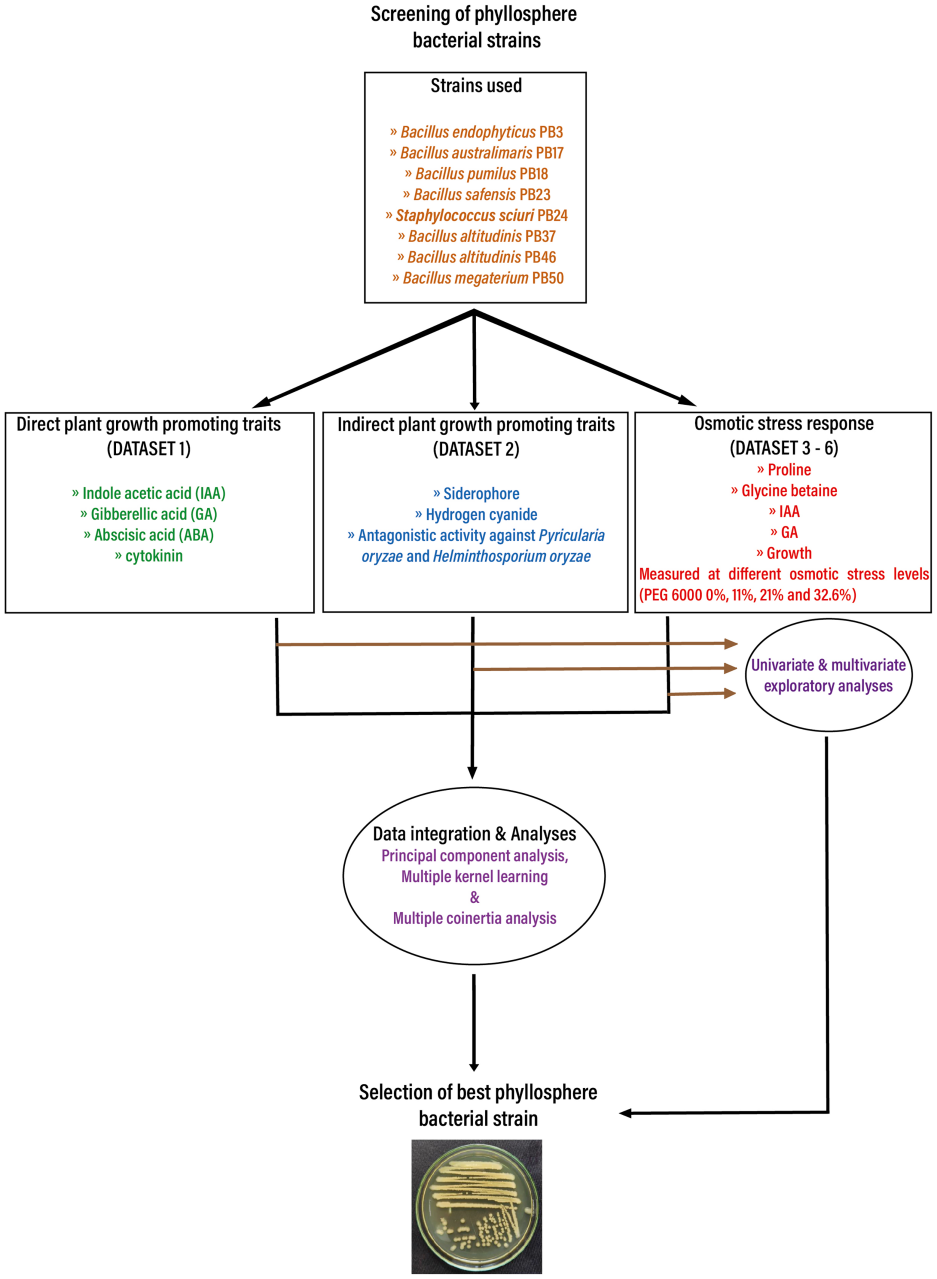
The flow chart depicts the basic workflow used in the current study to select the best phyllosphere bacterial strains for use in rice drought alleviation by screening the plant growth-promoting traits and osmotic stress response of bacterial strains. Obtained datasets were analyzed first using univariate and multivariate exploratory analyses. In the next step, the obtained datasets were integrated and analyzed using three unsupervised machine learning techniques: principal component analysis, multiple kernel learning, and multiple co-inertia analysis.

To assess PGP traits and osmotic stress responses in eight bacterial strains isolated from the phyllosphere of drought-tolerant rice varieties grown in Paramakudi, Tamil Nadu, India (Arun et al., 2020; Devarajan et al., 2021), two sets of different assessments were conducted. The following strains (genbank^1^ accession numbers in brackets) were included in this study: *Bacillus endophyticus* PB3 (MK969113), *B*. *australimaris* PB17 (MK979279), *B*. *pumilus* PB18 (MK979280), *B*. *safensis* PB23 (MK979280), *Staphylococcus sciuri* PB24 (MK994020), *B*. *altitudinis* PB37 (MK994020), *B*. *altitudinis* PB46 (MK979282), and *B*. *megaterium* PB50 (MK979284). The flowchart shows the study design in detail (Figure 1).

### Assessment of PGP traits

#### Production of phytohormones

To estimate the production of bacterial phytohormones, the strains were inoculated in triplicate into tryptic soy broth (TSB) and allowed to grow at 37°C for 24 h. The growth temperature for bacterial strains in TSB broth was set at 37°C for all studies. This temperature was selected based on the air temperature (42°C) in this region and the fact that the crop leaf surface temperature is expected to be 5°C lower (37°C) than the atmospheric temperature (Deva et al., 2020). Extraction, purification, and quantitative determination of IAA, GA, zeatin, and ABA from different strains were carried out using the methods described by Karadeniz et al. (2006). Briefly, phytohormones were extracted from the bacterial supernatant using ethyl acetate and thin-layer chromatography (TLC) was developed using a mixture of isopropanol/ammonia/distilled water (10:1:1 v/v/v). IAA, GA, zeatin, and ABA bands and R_f_ (retardation factor) values of the samples were visualized under 254 nm of UV light according to the standards of IAA, GA, zeatin, and ABA. The detected bands were scraped from TLC plates and dissolved in methanol. The purified samples were analyzed by ultra-high-performance liquid chromatography (UHPLC) using an evaporative light scattering detector (ELSD) system with reversed phase C-18 column (Shimadzu, Japan) at an isocratic flow rate of 0.5 min ml^−1^ at 40°C. The wavelengths were 280, 208, 254, and 265 nm for IAA, GA, zeatin, and ABA, respectively (HiMedia, India), and the total duration for the detection of each hormone was approximately 15 min. The results of all phytohormone concentrations are expressed as μg ml^−1.^

#### Production of siderophores

Siderophores production was evaluated in all bacterial strains. Briefly, the chrome azurol sulfonate (CAS) medium was prepared by adding the CAS solution to melted King’s B agar medium at a 1:15 ratio, and 10 μl of rice phyllosphere bacterial strains actively grown in TSB at 37°C for 24 h were spot inoculated on the center of the CAS plate. Colonies with a yellow-orange halo after 3 days of incubation (28 ± 2°C) were considered positive for siderophore production (Schwyn and Neilands, 1987). For siderophore quantification, 100 μl of fresh culture was inoculated into 100 ml of iron-free succinic broth medium and incubated in a rotary shaker at 30°C for 24 h (120 rpm). Subsequently, the broth culture was centrifuged at 10,000 rpm for 10 min. The absorbance of the supernatant was measured at 400 nm using a spectrophotometer (LAMBDA 365 UV–Vis spectrophotometer, PerkinElmer, Mumbai, India), and siderophore production was calculated using the molar extinction coefficient (ε = 20,000 M^−1^ cm^−1^). The hydroxamate- and catecholate-type siderophores were characterized using Arnow’s and tetrazolium tests, respectively (Arnow, 1937). The quantification of siderophores is represented as μM, and the presence or absence of siderophore type is expressed by the signs “+” and “−,” respectively.

#### Production of HCN

The production of HCN by the bacterial strains was measured using the alkaline picric acid method described by Wei et al. (1991). For the qualitative measurement of HCN production, a change in the color of the filter paper strips from yellow to light brown, brown, or brick red was recorded as a mild (+), moderate (+ +), or strong (+ + +) reaction, respectively, whereas no color shift was considered as a negative (−) reaction. The color in the paper was eluted using 10 ml of distilled water, and the filter paper in a sterile medium blank was used as a control. HCN quantification was performed by measuring the absorbance of the eluted samples at 625 nm, and the results are presented as optical density (OD) units.

#### Evaluation of antagonistic activity

The biocontrol activity of all bacterial strains was tested against two fungal pathogens (*Pyricularia oryzae* and *Helminthosporium oryzae*) obtained from the Department of Plant Pathology, Tamil Nadu Agricultural University, Coimbatore, India, following the protocol proposed by Ji et al. (2013). In brief, the mycelial disks (5 mm diameter) of each rice pathogenic fungus were placed on the edge of potato dextrose agar media (30 mm), and each bacterium was streak inoculated close to the center of the plates that were incubated for 5–6 days at 25°C. Subsequently, the mycelial growth inhibition percentage of *P*. *oryzae* (PO.IP) and *H*. *oryzae* (HO.IP) by each bacterial strain was calculated as follows:

Inhibition percentage = [(growth of pathogen in control − growth of pathogen with bacterial strains)/growth of pathogen in control] × 100

### Evaluation of osmotic stress effect on bacterial strains

#### Estimation of bacterial growth and sample preparation

Phyllosphere bacterial strains were inoculated in 100 ml of TSB in side-arm flasks with different concentrations of PEG 6000 (0, 11, 21, and 32.6%) and incubated at 37°C for 24 h. The optical density (OD) of the broth cultures was estimated at 600 nm, and the growth results were expressed as OD units. The cells were then centrifuged at 10, 000 rpm for 5 min and the supernatant was collected to assess IAA and GA production. Cell pellets were used to measure the accumulation of endogenous proline and GB.

#### Estimation of IAA and GA production

To determine IAA concentration, 2 ml of the supernatant was mixed with 4 ml of Salkowski reagent (2% of 0.5 M FeCl_3_ in 35% HClO_4_), and sterile TSB was used as a control (Meudt and Gaines, 1967). Subsequently, the mixtures were incubated in the dark at 25°C for 24 h. The absorbance of IAA was measured at 520 nm using a spectrophotometer. The concentration of IAA in the samples was determined using the IAA standard curve and expressed as μg ml^−1^.

To determine GA concentrations, 2 ml of the supernatant was mixed with 2 ml of zinc acetate solution and 2 ml of potassium ferrocyanide solution before centrifugation at 8,000 rpm for 10 min (Holbrook et al., 1961). A 5-ml aliquot of supernatant was added to 5 ml of 30% hydrochloric acid and incubated at 27°C for 75 min. The absorbance was measured at 254 nm, and the GA concentration in the samples was determined using a standard GA curve and expressed as μg ml^−1^.

#### Estimation of proline and GB production

The endogenous production of proline and GB was measured using the protocol described by Qurashi and Sabri (2013). The harvested cells were boiled and centrifuged to collect the supernatant. For proline estimation, 150 μl of the supernatant was mixed with 100 ml of water and 1 ml of ninhydrin reagent (0.35% ethanol), and 150 μl of sterile water was used as a control. This mixture was heated for 20 min, and then the absorbance was measured at 520 nm. The concentration of proline was calculated using a standard curve prepared with L-proline and expressed as μg ml^−1^.

To estimate the endogenous accumulation of GB, the extracted supernatant was diluted (1:1) in boiled 2 N H_2_SO_4_, and sterile water was used as a control (Qurashi and Sabri, 2013). The mixture (0.50 ml) was cooled for 60 min in ice water, and 200 μl of cold KI-I_2_ reagent was added with gentle vortexing. The mixture was incubated for 16 h at 4°C and gradually centrifuged for 15 min at 10,000 rpm. After carefully removing the supernatant, the resulting pellet was dissolved in 9 ml of 1,2-dichloroethane, and the absorbance was measured at 365 nm. The concentration of GB was calculated using a standard curve and expressed as μg ml^−1^.

### Statistical analysis

All experiments were conducted in triplicate, and the results are expressed as means with standard deviations. The obtained data were checked for normality and outliers and log-transformed prior to data analysis, if necessary. The following data analysis methods were used for analysis of direct and indirect microbial PGP traits: one-way ANOVA and multivariate ANOVA (MANOVA) with strain type as a single factor. Two-way ANOVA and MANOVA was applied to the set of microbial parameters (growth, production of indole-acetic acid, gibberellic acid, proline, and glycine betaine) with two factors—strain type and stress level. One-way ANOVA test was followed by Tukey’s post-hoc test. The significance level was set to 0.05 for all tests. Multivariate exploratory analyses included principal component analysis (PCA), heatmaps, k-means clustering, and spectral clustering. PCA was performed separately for two datasets (direct and indirect PGP traits and osmotic stress data). Heatmaps, k-means clustering, and spectral clustering were used as additional methods for analysis of osmotic stress dataset. The details of software packages used for the data analysis are provided in Supplementary Table S1 (Paradis et al., 2004; Lê et al., 2008; R Core Team, 2013; Meng et al., 2014; Mariette and Villa-Vialaneix, 2018; Kolde, 2019; John et al., 2020). In the case of PCA, the variables contributing significantly to the principal component (PC) axes were determined using the PCAtest package with random permutation, a bootstrap replication value of 1,000, and an alpha level of 0.05 (Camargo, 2022). The importance of the variable was ranked from each principal component axis using the total loading values.

### Data integration

We used horizontal integration of the datasets to compare multiple treatment factors on similar variables and samples. We applied unsupervised machine learning techniques and implemented them using three integration techniques: early integration (PCA), mixed integration (multiple kernel learning, MKL), and intermediate integration (multiple co-inertia analysis, MCIA). Supplementary Table S10 contains information on the dataset combinations used for the integration methods.

In the case of the PCA analysis, the datasets were first combined and transformed, and then the PCA function from the factomineR package was applied to the obtained dataset (Supplementary Table S10). MCIA is a mixed integration technique in which the dataset is dimensionally reduced and transformed using ordination methods, such as PCA, correspondence analysis (COA), or non-symmetric correspondence analysis (NSCA), before being combined for analysis. In this study, the MCIA was performed on the data list (Supplementary Table S10) using the omicade4 package (Supplementary Table S1). Data dimension reduction, transformation, and analysis were performed using a single-function MCIA with singular value decomposition. The output was saved as a MCIA class object, and from that object, the variables from each dataset were visualized with regard to the sample relationship using the plotVar function.

The same data list was used in the R package mixKernel to perform MKL (Supplementary Tables S1, S10), wherein each dataset was first converted into a kernel object using the compute.kernel function with a linear kernel, thereby converting a linearly inseparable space object into a linearly separable one. The fully unsupervised MKL (UKML) method was used to combine all kernel objects, and the resulting kernels were subjected to PCA and an important variable analysis using the kernel.pca and kernel.pca.permute functions, respectively.

The results of different data integration techniques were compared using the congruence among distance matrices (CADM) approach (Campbell et al., 2011) implemented in the CADM.global function using the R package ape: analyses of phylogenetics and evolution (Supplementary Table S1).

## Results

### Direct and indirect PGP activities of phyllosphere bacterial strains

The analysis of direct PGP activities showed significant differences (one-way MANOVA, *p* < 0.001) in the production of phytohormones by the studied bacterial strains (Table 1; Supplementary Table S2). *Bacillus megaterium* PB50 showed the highest production of all four phytohormones, followed by *B*. *endophyticus* PB3, which produced the highest levels of IAA, GA, and ABA, and *B*. *altitudinis* PB46, which produced high levels of GA and cytokinin. The lowest levels of IAA and GA were produced by *B*. *australimaris* PB17 and those of cytokinin and ABA were produced by *B*. *safensis* PB23 and *S*. *sciuri* PB24, respectively.

**Table 1:** Mean and standard deviation values of the direct plant growth-promoting traits [indole-acetic acid (IAA), gibberellic acid (GA), cytokinin, and abscisic acid (ABA) production] measured for each rice phyllosphere bacterial strain (number of replicates, *n* = 3).

Strains	IAA	GA	Cytokinin	ABA
	(μg ml^−1^)	(μg ml^−1^)	(ng ml^−1^)	(ng ml^−1^)
*Bacillus endophyticus* PB3	32.5 (0.73)^b^	34.8 (1.2)^b^	148.4 (4.1)^c^	239.8 (7.8)^b^
*B*. *australimaris* PB17	7.57 (0.37)^g^	9.2 (0.3)^f^	63.3 (1.5)^e^	74.5 (2.3)^f^
*B*. *pumilus* PB18	15.1 (0.39)^f^	14.7 (0.4)^d^	78.2 (0.6)^d^	92.8 (3.4)^e^
*B*. *safensis* PB23	20.4 (0.32)^d^	19.8 (0.4)^c^	28.9 (0.5)^g^	59.3 (1.6)^g^
*Staphylococcus sciuri* PB24	18.4 (0.44)^e^	13.8 (0.4)^d^	52.6 (1.9)^f^	34.4 (3.3)^h^
*B*. *altitudinis* PB37	19.5 (0.46)^de^	10.3 (0.4)^e^	80.2 (0.9)^d^	124.4 (3.6)^d^
*B*. *altitudinis* PB46	26.4 (0.35)^c^	35.7 (0.3)^b^	178.1 (1.0)^b^	147.2 (2.0)^c^
*B*. *megaterium* PB50	38.9 (0.86)^a^	46.6 (0.4)^a^	410 (5.2)^a^	311.7 (30.7)^a^

Parameter values with different letters are significantly different according to Tukey’s test, *p* < 0.05.

Similarly, the studied strains significantly differed in their indirect PGP trait values (one-way MANOVA, *p* < 0.001, Table 2; Supplementary Table S2). Siderophore production was found to be the highest in *B*. *megaterium* PB50, followed by *B*. *pumilus* PB18. The characterization of siderophores revealed that only *B*. *megaterium* PB50 produced hydroxamate-type siderophores, whereas the remaining strains produced catecholate-type siderophores (Table 2). The analysis of HCN production results showed that *B*. *pumilus* PB18 produced the highest amount of HCN, while *B*. *safensis* PB23 and *S*. *Sciuri* PB24 produced the least amount of HCN. The assessment of the antagonistic activity of phyllosphere bacterial strains against the rice foliar pathogens revealed that only *B*. *endophyticus* PB3 and *B*. *australimaris* PB17 had an antagonistic activity against *P*. *oryzae*, and only *B*. *megaterium* PB50 inhibited the growth of *H*. *oryzae* (Table 2; Supplementary Figure S1).

**Table 2:** Mean and standard deviation values of the indirect plant growth-promoting traits, such as siderophore (Sid.) production, qualitative (Qual.), and quantitative (Quant.) assessment of hydrogen cyanide (HCN) production, and growth inhibition of *Pyricularia oryzae* and *Helminthosporium oryzae* measured for each rice phyllosphere bacterial strain (number of replicates, *n* = 3).

Strains	Sid. production (μM)	Arnow’s test	Tetrazolium test	Qual. HCN production	Quant. HCN production (OD_625_)	Inhibition percentage (%)	
						*Pyricularia oryzae*	*Helminthosporium oryzae*
*Bacillus endophyticus* PB3	46.1 (2.2)^c^	+	−	**++**	0.083 (0.002)^b^	71.2 (4.2)^b^	0
*B*. *australimaris* PB17	38.4 (1.5)^d^	+	−	**++**	0.062 (0.004)^c^	0	0
*B*. *pumilus* PB18	65.5 (1.5)^b^	+	−	**+++**	0.128 (0.003)^a^	79.7 (2.7)^a^	0
*B*. *safensis* PB23	14.8 (0.6)^f^	+	−	**−**	0.013 (0.003)^f^	0	0
*Staphylococcus sciuri* PB24	37.1 (0.6)^d^	+	−	**−**	0.009 (0.001)^f^	0	0
*B*. *altitudinis* PB37	17.3 (0.5)^f^	+	−	**+**	0.041 (0.003)^e^	0	0
*B*. *altitudinis* PB46	20.7 (0.8)^e^	+	−	**++**	0.089 (0.004)^b^	0	0
*B*. *megaterium* PB50	72.2 (3.2)^a^	−	+	**+**	0.049 (0.004)^d^	0	44.4 (5.2)^a^

Parameter values with different letters are significantly different according to Tukey’s test, *p* < 0.05.

### Bacterial growth and production of phytohormones and osmolytes under different osmotic stress levels

**Figure 2:**
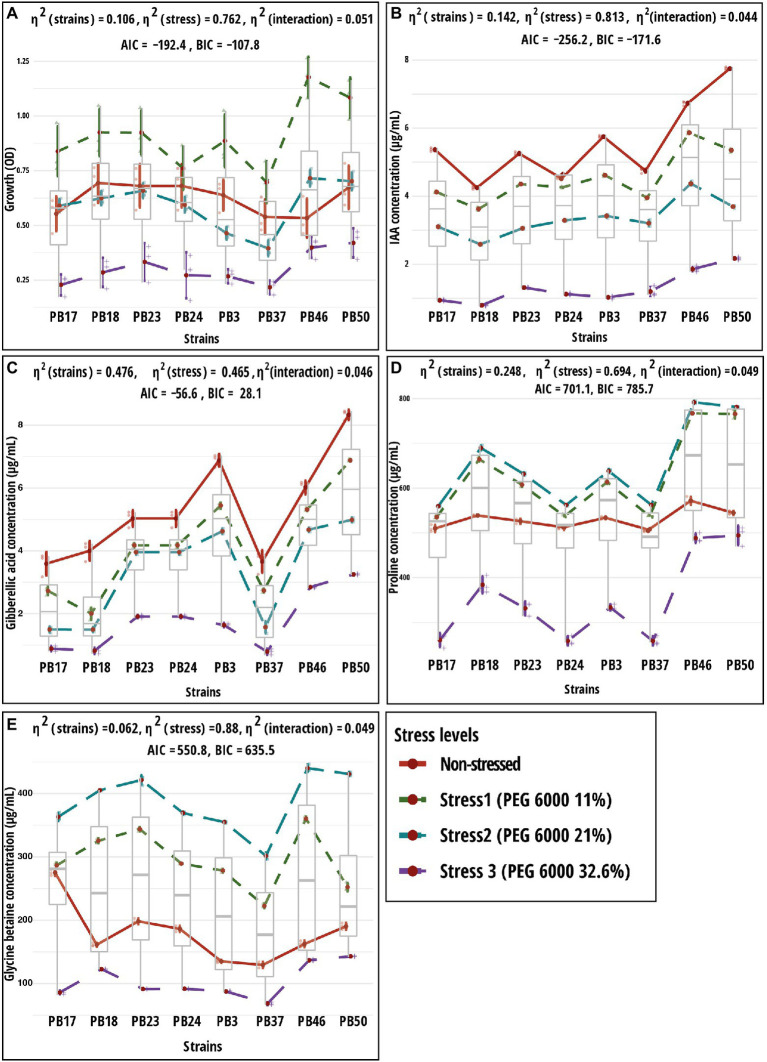
Results of two-way ANOVA showing the mean differences of dependent variables according to two independent variables (strain and stress) and their interaction. Shown are mean and standard error values for two grouping variables. Plots **(A)** growth; **(B)** indole-acetic acid (IAA); **(C)** gibberellic acid (GA); **(D)** proline; and **(E)** glycine betaine. The strain codes refer to the following strains: *Bacillus endophyticus* PB3, *B*. *australimaris* PB17, *B*. *pumilus* PB18, *B*. *safensis* PB23, *S*. *sciuri* PB24, *B*. *altitudinis* PB37, *B*. *altitudinis* PB46, and *B*. *megaterium* PB50. AIC, akaike information criterion; BIC, Bayesian information criterion; and η^2^, effect size. Individual *p* values are given in Supplementary Table S5.

Analysis of bacterial growth under different osmotic stress levels showed that under non-stressed conditions, *B*. *pumilus* PB18, *B*. *safensis* PB23, *S*. *sciuri* PB24, and *B*. *megaterium* PB50 reached the highest OD, with no significant difference between them. No significant difference was observed among *B*. *australimaris* PB17, *B*. *altitudinis* PB37, and *B*. *altitudinis* PB46, which had the lowest OD values (Table 3; Supplementary Table S6). The maximum growth values were achieved by *B*. *altitudinis* PB46 at 11 and 21% PEG 6000 concentrations and by *B*. *megaterium* PB50 at 32% PEG 6000 (Figure 2A). The lowest growth was observed in *B*. *altitudinis* PB37 at 11 and 21% PEG 6000 concentrations and in *B*. *australimaris* PB17 and *B*. *altitudinis* PB37 at 32.6% PEG 6000, with no significant difference between them.

**Table 3:** Mean and standard deviation values of growth of each rice phyllosphere bacterial strain (number of replicates, *n* = 3) measured under three different osmotic stress conditions and a non-stress condition.

Strains	Growth (OD)			
	Non-stress	PEG 6000 (11%)	PEG 6000 (21%)	PEG 6000 (32.6%)
*Bacillus endophyticus* PB3	0.638 (0.007)^b^	0.886 (0.01)^d^	0.464 (0.003)^f^	0.267 (0.003)^e^
*B*. *australimaris* PB17	0.553 (0.008)^c^	0.838 (0.01)^e^	0.590 (0.003)^e^	0.228 (0.005)^f^
*B*. *pumilus* PB18	0.693 (0.009)^a^	0.924 (0.01)^c^	0.624 (0.003)^d^	0.285 (0.007)^d^
*B*. *safensis* PB23	0.680 (0.01)^a^	0.924 (0.01)^c^	0.658 (0.003)^c^	0.333 (0.009)^c^
*Staphylococcus sciuri* PB24	0.681 (0.01)^a^	0.760 (0.01)^f^	0.594 (0.003)^e^	0.272 (0.01)^de^
*B*. *altitudinis* PB37	0.539 (0.008)^c^	0.696 (0.01)^g^	0.395 (0.004)^g^	0.217 (0.003)^f^
*B*. *altitudinis* PB46	0.533 (0.009)^c^	1.177 (0.01)^a^	0.715 (0.004)^a^	0.399 (0.005)^b^
*B*. *megaterium* PB50	0.673 (0.01)^a^	1.083 (0.01)^b^	0.701 (0.004)^b^	0.420 (0.007)^a^

Parameter values with different letters are significantly different according to Tukey’s test, *p* < 0.05.

As the PEG 6000 concentration increased, IAA and GA production decreased in all strains (Table 4; Figures 2B,C). The strain *B*. *megaterium* PB50 produced the highest IAA amount under almost all stress conditions, except for PEG 6000 at the 21% level, when this value was the highest for *B*. *altitudinis* PB46. The strain *B*. *megaterium* PB50 also exceeded the other studied strains in the production of GA at all stress levels, followed by *B*. *endophyticus* PB3 at 11% PEG 6000 and *B*. *altitudinis* PB46 at 21 and 32.6% PEG 6000.

**Table 4:** Mean and standard deviation values of indole-acetic acid (IAA) and gibberellic acid (GA) production (μg ml^−1^) by each rice phyllosphere bacterial strain (number of replicates, n = 3) measured under three different osmotic stress conditions and a non-stress condition.

Strains	Non-stress		PEG 6000 (11%)		PEG 6000 (21%)		PEG 6000 (32.6%)	
	IAA	GA	IAA	GA	IAA	GA	IAA	GA
*Bacillus endophyticus* PB3	33.0 (0.4)^c^	47.0 (0.8)^b^	21.3 (0.4)^c^	31.4 (0.42)^b^	11.7 (0.4)^c^	20.7 (0.17)^b^	1.07 (0.08)^ef^	2.47 (0.77)^c^
*B*. *australimaris* PB17	28.8 (0.6)^d^	12.6 (1.7)^g^	17.0 (0.2)^e^	8.99 (0.17)^e^	9.7 (0.3)^e^	1.93 (0.17)^e^	0.89 (0.04)^fg^	0.64 (0.09)^d^
*B*. *pumilus* PB18	18.1 (0.4)^f^	15.9 (1.0)^f^	13.2 (0.4)^g^	5.87 (0.51)^f^	6.7 (0.2)^f^	1.23 (0.18)^f^	0.63 (0.03)^g^	0.52 (0.18)^d^
*B*. *safensis* PB23	27.6 (0.6)^d^	25.0 (0.5)^d^	19.0 (0.2)^d^	19.3 (0.49)^d^	9.4 (0.2)^e^	15.0 (0.29)^c^	1.74 (0.03)^c^	3.46 (0.26)^c^
*Staphylococcus sciuri* PB24	17.4 (0.3)^f^	22.1 (1.2)^e^	18.2 (0.3)^de^	5.5 (0.34)^f^	10.9 (0.2)^cd^	3.1 (0.34)^d^	1.27 (0.06)^de^	0.86 (0.23)^d^
*B*. *altitudinis* PB37	22.6 (0.7)^e^	13.1 (0.3)^fg^	15.7 (0.4)^f^	9.31 (0.17)^e^	10.3 (0.6)^de^	1.86 (0.17)^e^	1.46 (0.36)^cd^	0.47 (0.25)^d^
*B*. *altitudinis* PB46	45.3 (0.9)^b^	35.9 (0.9)^c^	34.4 (0.6)^b^	29.9 (0.93)^c^	19.1 (0.6)^a^	21.2 (0.34)^b^	3.46 (0.30)^b^	7.93 (0.13)^b^
*B*. *megaterium* PB50	60.1 (0.7)^a^	69.0 (0.9)^a^	28.6 (0.7)^a^	49.0 (0.45)^a^	13.7 (0.3)^b^	24.3 (0.51)^a^	4.72 (0.16)^a^	10.4 (0.63)^a^

Parameter values with different letters are significantly different according to Tukey’s test, *p* < 0.05.

All studied strains showed a gradual increase in proline and GB production from non-stress to stress conditions at 21% PEG 6000, but at 32.6% PEG 6000, the production dropped below the non-stress level for both osmolytes (Table 5; Figures 2D,E). Nevertheless, substantial differences were observed for the different strains in osmolyte production at different stress levels. *Bacillus pumilus* PB18 accumulated the highest proline content under non-stress conditions, whereas *B*. *megaterium* PB50 accumulated the lowest amount under these conditions. Furthermore, under 11, 21, and 32.6% PEG 6000 concentrations, the bacterial strains *B*. *pumilus* PB18 and *B*. *safensis* accumulated the maximum amount of proline, with no significant difference between them. The strains *B*. *australimaris* PB17 and *B*. *altitudinis* PB37 accumulated the lowest amount of proline at all three stress levels, with no significant differences between them. Different strains showed different GB production patterns under variable stress conditions. Under non-stress conditions, the bacterial strain *B*. *australimaris* PB17 produced the highest GB, whereas *B*. *altitudinis* PB46 and *B*. *megaterium* PB50 produced the highest GB at 11 and 21% PEG 6000 levels. *Bacillus megaterium* PB50 produced the maximum GB at 32.6% PEG 6000. Under non-stress conditions as well as 11, 21, and 32.6% PEG 6000 levels, *B*. *altitudinis* PB37 had the lowest GB.

**Table 5:** Mean and standard deviation values of proline and glycine betaine (GB) accumulation (μg ml^−1^) by each rice phyllosphere bacterial strain (number of replicates, *n* = 3) measured under three different osmotic stress conditions and a non-stress condition.

Strains	Non-stressed		PEG 6000 (11%)		PEG 6000 (21%)		PEG 6000 (32.6%)	
	Proline	GB	Proline	GB	Proline	GB	Proline	GB
*Bacillus endophyticus* PB3	534 (15)^ab^	135 (7)^c^	614 (19)^c^	278 (20)^d^	638 (22)^c^	354 (18)^c^	334 (14)^c^	89 (5)^e^
*B*. *australimaris* PB17	510 (16)^b^	275 (18)^a^	535 (17)^e^	287 (11)^d^	560 (19)^d^	363 (15)^c^	261 (9)^d^	86 (5)^e^
*B*. *pumilus* PB18	571 (15)^a^	162 (12)^bc^	767 (14)^a^	325 (9)^c^	792 (15)^a^	405 (23)^bc^	488 (16)^a^	123 (5)^c^
*B*. *safensis* PB23	541 (13)^ab^	190 (18)^b^	766 (24)^a^	343 (13)^ab^	781 (17)^a^	421 (19)^ab^	494 (18)^a^	91 (5)^d^
*Staphylococcus sciuri* PB24	526 (18)^b^	186 (14)^b^	608 (25)^c^	289 (19)^d^	632 (32)^c^	369 (21)^c^	332 (12)^c^	92 (3)^d^
*B*. *altitudinis* PB37	534 (10)^ab^	129 (23)^c^	537 (13)^e^	223 (7)^e^	562 (11)^d^	301 (12)^d^	259 (6)^d^	68 (5)^f^
*B*. *altitudinis* PB46	539 (15)^ab^	162 (8)^bc^	664 (24)^b^	360 (9)^a^	690 (29)^b^	440 (15)^a^	384 (11)^b^	137 (5)^b^
*B*. *megaterium* PB50	384 (14)^c^	198 (11)^b^	585 (22)^d^	352 (12)^a^	610 (24)^cd^	431 (17)^a^	313 (18)^c^	143 (4)^a^

Parameter values with different letters are significantly different according to Tukey’s test, *p* < 0.05.

Two-way MANOVA revealed the significance of the interaction effect (strain–osmotic stress interaction) and the main effects (strains and osmotic stress level) in the examined parameters (Supplementary Table S3). The results of one-way MANOVA revealed that the impact of bacterial strains on microbial parameters was statistically significant at each stress level (Supplementary Table S4), except for bacterial growth, which was not significant in the non-stress conditions (Supplementary Tables S6–S9). The two-way ANOVA results showed significant (*p* < 0.05) differences in all traits tested among bacterial strains and osmotic stress levels, as well as their interaction effects (Supplementary Table S5).

**Figure 3:**
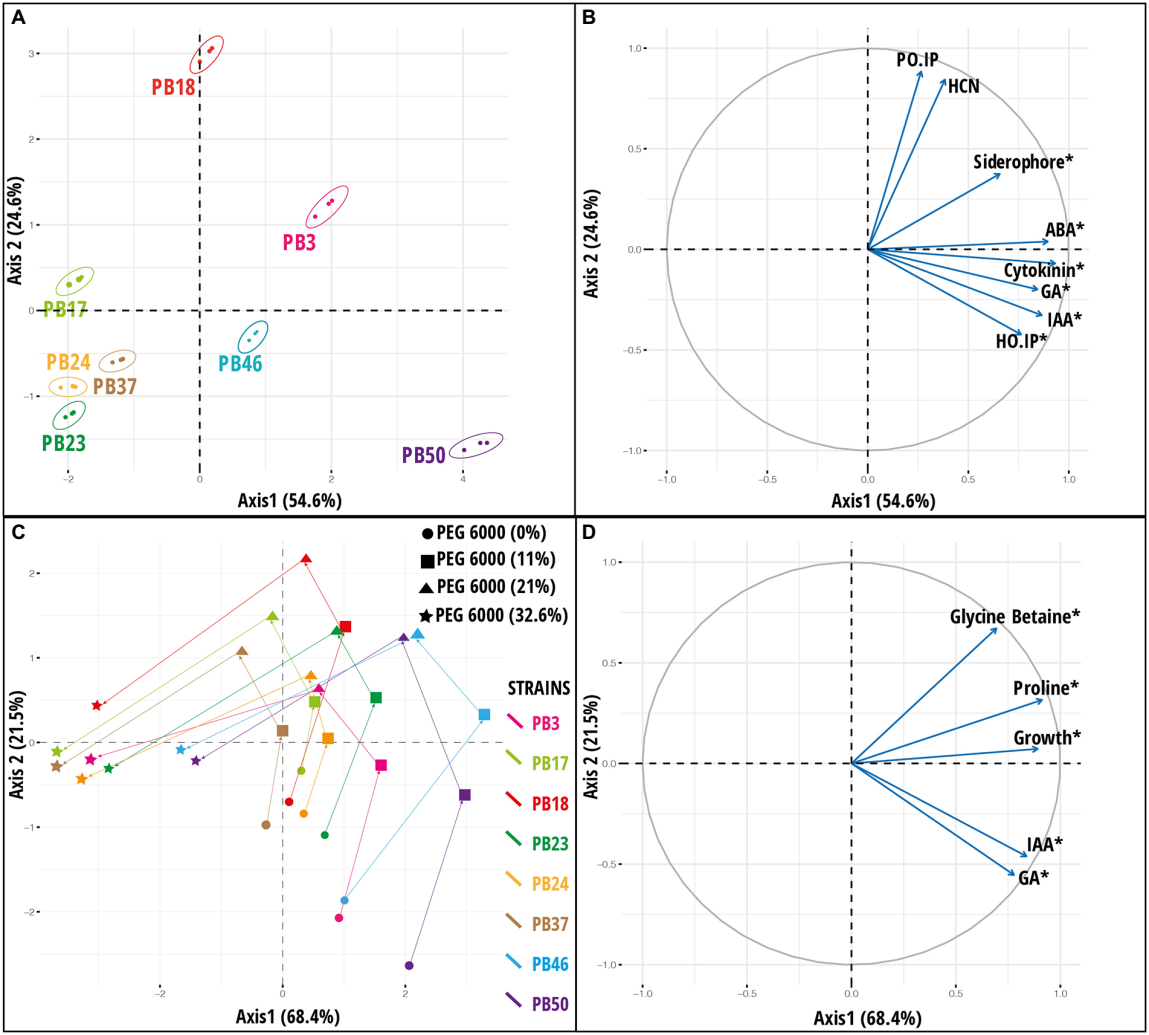
Results of principal component analysis (PCA) based on the plant growth-promoting (PGP) trait data of rice phyllosphere bacteria (number of replicates, *n* = 3). **(A)** Score plot and **(B)** loading plot of variables according to first two principal component axes. Bacterial strains are indicated by 95% confidence ellipses. The plots correspond to 79.2% of the total data variance and variance proportions are shown along each principal component axis. **(C)** Score plot and **(D)** loading plot of variables along first two principal component axes based on microbial dataset [indoleacetic acid (IAA), gibberellic acid (GA), proline, glycine betaine, and growth] of rice phyllosphere bacterial strains measured at different PEG 6000 concentrations (number of replicates, *n* = 3). The plots correspond to 89.9% of the total data variance, and variance proportions are shown along each principal component axis. Variables with asterisk in plot B and D are significant along the first principal component axis. Abbreviations used in plot **(B)** are, abscisic acid (ABA), hydrogen cyanide (HCN), *Helminthosporium oryzae* inhibition percentage (HO_IP), and *Pyricularia oryzae* inhibition percentage (PO_IP). The codes of the strains in plots **(A,C)** refer to the following strains: *Bacillus endophyticus* PB3, *B*. *australimaris* PB17, *B*. *pumilus* PB18, *B*. *safensis* PB23, *Staphylococcus sciuri* PB24, *B*. *altitudinis* PB37, *B*. *altitudinis* PB46, and *B*. *megaterium* PB50.

The response pattern of the studied strains based on biochemical properties and growth under various osmotic stress levels was revealed by the PCA plot (Figure 3). The PCA first (PC1) axis captured 68.4% of the overall data variation, and all variables significantly contributed to the PCA first axis (Supplementary Table S11). All strains had a generally similar response pattern that consisted of a gradual change along the PCA second (PC2) axis in the case of 11 and 21% stress levels of PEG 6000, followed by a shift in the same direction along the PC1 axis when strains were exposed to 32.6% PEG 6000 (Figure 3C). The gradual change along the PC2 axis was related mainly to the increase in GB and proline production with the increase in PEG 6000 level to 21%, while the application of 32.6% PEG 6000 was characterized by a reduction in strain growth values (Figure 3D).

The heatmap based on PGP traits showed that the strains could be divided into two main groups based on their response to osmotic stress levels. At the 32% PEG 6000 level, the strains formed one group, and the strains at other stress levels formed another cluster (Supplementary Figure S4). The results of *k*-means clustering indicated four optimal clusters, of which two (stress levels 0 and 32.6%) were partially homogenous (Supplementary Figure S5 A). Moreover, these results indicated that *B*. *altitudinis* PB46 and *B*. *megaterium* PB50 were the most deviant in their responses to different stress levels. Spectral clustering identified two strain groups with similar response patterns to increasing PEG 6000 stress levels. The first group consisted of four strains (*B*. *endophyticus* PB3, *B*. *pumilus* PB18, *B*. *altitudinis* PB46, and *B*. *megaterium* PB50), and the second group consisted of three strains (*B*. *australimaris* PB17, *B*. *safensis* PB23, and *S*. *sciuri* PB24; Supplementary Figure S5 B). *Bacillus altitudinis* PB37 was not included in either group. The main difference between the response dynamics of the two groups was related to a more profound stress response in the first group than in the second group and the strain *B*. *altitudinis* PB37. This higher stress response was more reflected at the stress levels of 11 and 21% PEG 6000 when the relative stress response magnitude was 1.5–2-fold higher in the first group than in the second group.

### Integration of different datasets

#### Early integration approach

A PCA was used to explore the variation among strains based on a joint dataset of direct and indirect PGP variables (Figure 3). The PC1 axis explained 54.6% of the data variance, and the statistically significant variables in PCA were siderophore, IAA, GA, GB, ABA production, and *H*. *oryzae* inhibition percentage (Supplementary Table S11). The PC1 axis separated *B*. *megaterium* PB50, *B*. *altitudinis* PB46, and *B*. *endophyticus* PB3 from the other strains (Figures 3A,B). The PC2 axis emphasized the variation among strains in *P*. *oryzae* inhibition percentage and HCN production ability, indicating that strain *B*. *pumilus* PB18 exhibited the highest *P*. *oryzae* inhibition percentage and HCN production potential among the studied strains. A heatmap based on direct and indirect PGP traits was used to assess similarities among the studied bacterial strains (Supplementary Figure S2). Based on the intensity of the PGP response, the strains were separated into one large group of seven strains with two subclusters, whereas the *B*. *megaterium* PB50 strain formed a separate cluster. The large cluster comprised two sub-clusters, with *B*. *endophyticus* PB3, *B*. *pumilus* PB18, and *B*. *altitudinis* PB46 grouped together as moderate-performance strains, and *B*. *australimaris* PB17, *B*. *safensis* PB23, *S*. *sciuri* PB24, and *B*. *altitudinis* PB37 grouped separately because of their poor performance in PGP activities. Furthermore, *k*-means clustering led to the identification of six optimal clusters in this dataset, with *B*. *australimaris* PB17 and *B*. *altitudinis* PB37 and *B*. *safensis* PB23 and *S*. *sciuri* PB24 clustered together, and both clusters were close to each other, as shown in the PCA ordination plot (Supplementary Figure S3).

**Figure 4:**
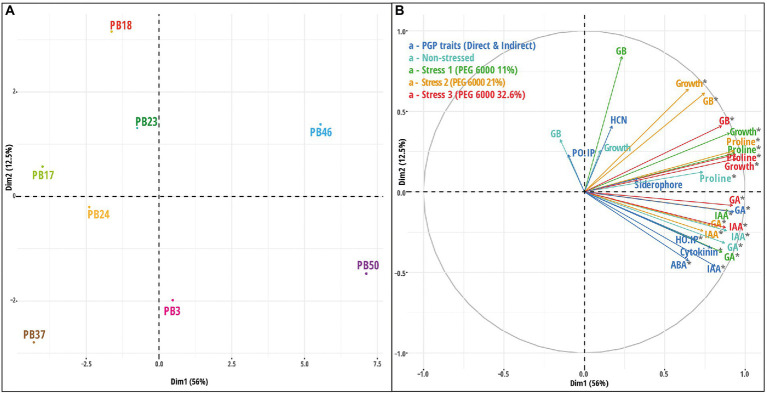
Results of principal component analysis (PCA) based on the integration of plant growth-promoting traits data and osmotic stress response parameters datasets. **(A)** Score plot and **(B)** loading plot of variables according to first two principal components. The plots correspond to 68.5% of the total data variance, and variance proportions are shown along each principal component axis. Variables with asterisk in the plot **(B)** are significant along the first principal component axis. Abbreviations used in plot **(B)** are, indoleacetic acid (IAA), gibberellic acid (GA), glycine betaine (GB), abscisic acid (ABA), hydrogen cyanide (HCN), *Helminthosporium oryzae* inhibition percentage (HO.IP), and *Pyricularia oryzae* inhibition percentage (PO.IP). The codes of the strains in plot **(A)** refer to the following strains: *Bacillus endophyticus* PB3, *B*. *australimaris* PB17, *B*. *pumilus* PB18, *B*. *safensis* PB23, *Staphylococcus sciuri* PB24, *B*. *altitudinis* PB37, *B*. *altitudinis* PB46, and *B*. *megaterium* PB50.

In addition to PCA on the joint PGP trait dataset (Figures 3A,B), PCA was performed by combining all osmotic stress datasets (datasets 3–6; Supplementary Table S10) and all six datasets (datasets 1–6; Supplementary Table S10). The results for PCA on all osmotic stress datasets are provided in Supplementary Figure S6, and these data were used as input later in the congruence among the distance matrices approach. The PCA results for all six datasets are shown in Figure 4. PC1 was significant (*p* < 0.05), accounting for 56% of the total variance, whereas PC2 accounted for only 12.5% of the total variance. Most variables had a significant impact on PC1 (Supplementary Table S11). The maximum variance was attributed by the variables measured at a stress level of 32.6% PEG 6000 32.6%.

#### Multiple co-inertia analysis

**Figure 5:**
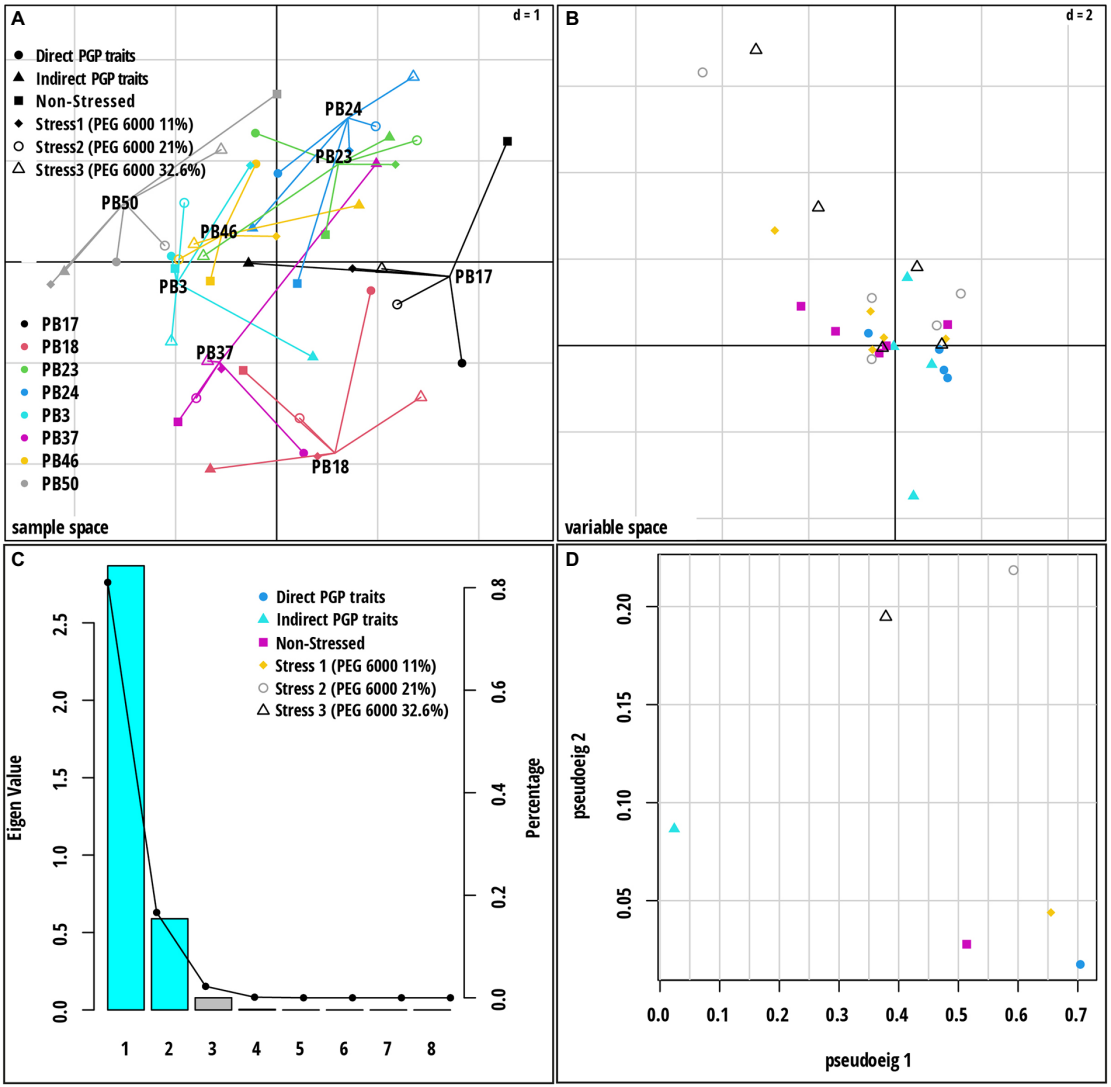
Multiple co-inertia analysis (MCIA) results based on six datasets [direct plant growth-promoting (PGP) traits, indirect PGP traits, microbial parameters under osmotic stress at PEG concentrations 0, 11, 21, and 32.6%]. **(A)** A plot of the first two components in the sample space. Each sample is represented by a shape where lines connect the six datasets for each sample to a center point (MCIA global score). **(B)** Variable space for each dataset. **(C)** A scree plot of absolute eigenvalues (bars) and the proportions of variance for the eigenvectors (line). **(D)** A plot of data weighting space that shows the pseudo-eigenvalues space of all datasets indicating the variance of an eigenvalue contributed by each dataset. The codes of the strains in plot **(A)** refer to the following strains: *Bacillus endophyticus* PB3, *B*. *australimaris* PB17, *B*. *pumilus* PB18, *B*. *safensis* PB23, *Staphylococcus sciuri* PB24, *B*. *altitudinis* PB37, *B*. *altitudinis* PB46, and *B*. *megaterium* PB50.

Multiple co-inertia analysis was applied to jointly analyze the six datasets (Supplementary Table S10). The graphical outputs of this analysis are shown in Figure 5; Supplementary Figure S7. The MCIA first axis captured the highest variance (78.3%) in the datasets and separated three strains, *B*. *endophyticus* PB3, *B*. *altitudinis* PB46, and *B*. *megaterium* PB50, from the remaining strains (Figure 5A). The MCIA second axis, explaining 16.0% of the data variation, emphasized the distinction between the strains *B*. *pumilus* PB18 and *B*. *altitudinis* PB37 and the other studied strains. The pseudo-eigenvalue space of six datasets (Figure 5D) indicates that three datasets (direct PGP traits as well as stress at PEG 6000 levels of 0 and 11%) contributed the most to the MCIA first axis, while the contribution of indirect PGP parameters was small. The datasets of the stress at PEG 6000 levels of 21 and 32.6% contributed to the MCIA second axis. Correlations between datasets were the highest at the stress levels of 11, 21, and 32.6% PEG 6000 (RV = 0.71–0.76) and lowest in the case of the datasets of indirect PGP traits and stress levels of 0, 11, and 21% of PEG 6000 (RV = 0.35–0.51). Projections of all variables onto the first two MCIA axes space indicated that the strains *B*. *altitudinis* PB46 and *B*. *megaterium* PB50, and to a lesser extent, *B*. *endophyticus* PB3, were associated with higher IAA and GA values (Supplementary Figure S7). The concentration of GB was an important variable for the separation of strains under non-stress conditions, and it coincided with higher siderophore and cytokinin production. The dataset correlation plot based on the RV coefficient value showed that the dataset of intermediate stress level (21% PEG 6000) was highly positively correlated with the datasets of 11 and 32.6% PEG 6000 datasets (Supplementary Figure S8 A). The positive correlation of the stress and non-stress datasets of 11% PEG 6000 with the datasets of PGP traits was higher than that of the other two stress levels, indicating that the stress level influenced the function of all studied strains. Indirect PGP traits did not positively correlate with other dataset types.

#### Multiple kernel learning

**Figure 6:**
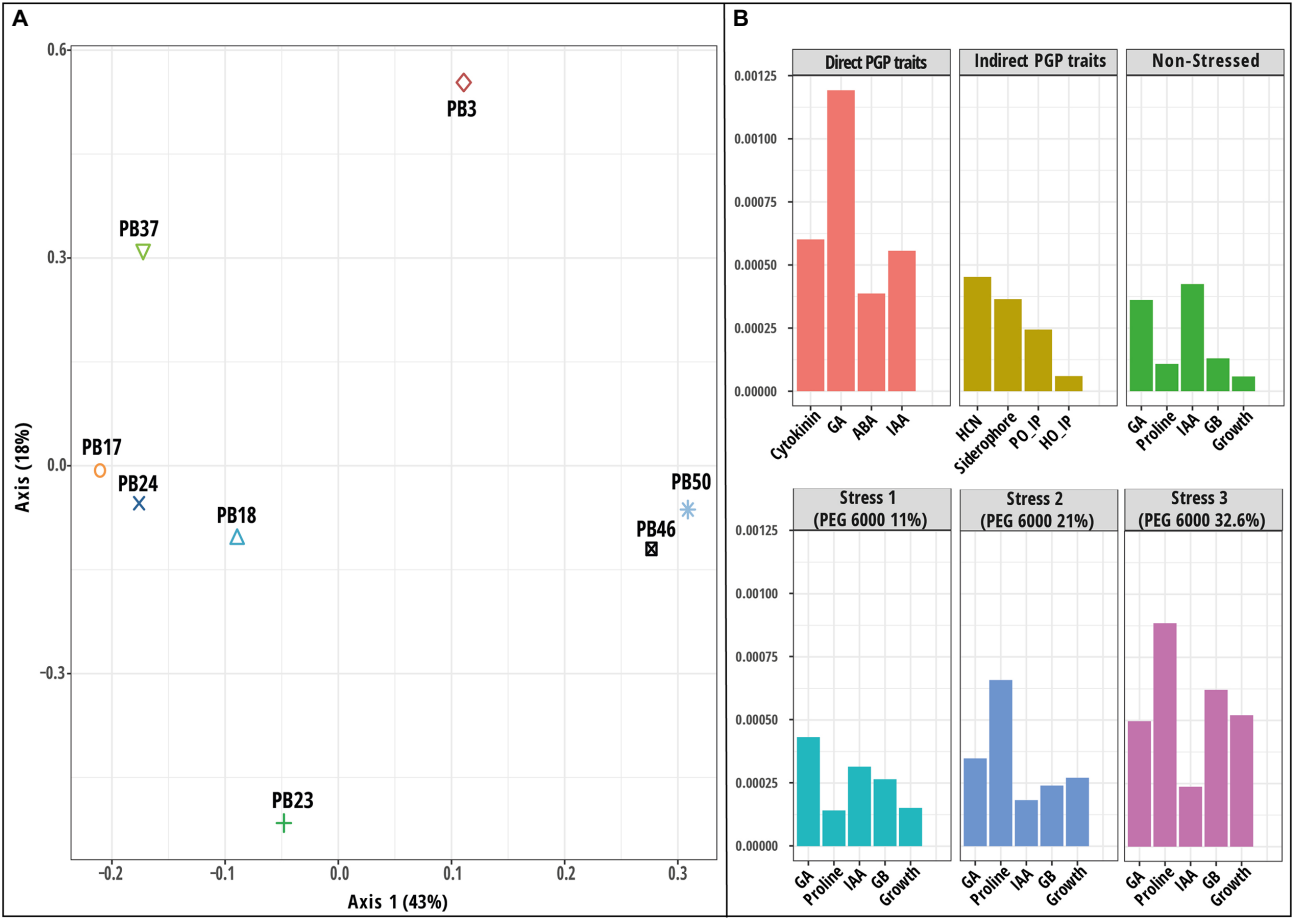
Results of multiple kernel learning analysis (MKL). **(A)** A plot of kernel principal component analysis (KPCA) based on six datasets (direct plant growth-promoting (PGP) traits, indirect PGP traits, microbial parameters under osmotic stress at polyethylene glycol (PEG) concentrations 0, 11, 21, and 32.6%). **(B)** A plot for important variables in each dataset assessed using the Crone-Crosby distance. The codes of the strains in plot **(A)** refer to the following strains: *Bacillus endophyticus* PB3, *B*. *australimaris* PB17, *B*. *pumilus* PB18, *B*. *safensis* PB23, *Staphylococcus sciuri* PB24, *B*. *altitudinis* PB37, *B*. *altitudinis* PB46, and *B*. *megaterium* PB50. Abbreviations used in plot **(B)** are indoleacetic acid (IAA), gibberellic acid (GA), abscisic acid (ABA), hydrogen cyanide (HCN), *Helminthosporium oryzae* inhibition percentage (HO_IP), and *Pyricularia oryzae* inhibition percentage (PO_IP).

The MKL technique was applied to the same six datasets (Supplementary Table S10), and the combined kernel principal component analysis (KPCA) was used in further exploratory analysis. The results of these analyses revealed that the majority of the overall variance in the data was captured by the first axis of KPCA, which clearly separated the two strains, *B*. *altitudinis* PB46 and *B*. *megaterium* PB50, from the rest of the strains (Figure 6A). To identify the influence of the variables on KPCA, an important variable plot was computed (Figure 6B), which showed that GA and HCN production were the most important direct and indirect PGP traits, respectively. The production of IAA and GA was important in the non-stress conditions, whereas GA was also relevant at the 11% PEG 6000 stress level. Proline production was found to be an important variable in both 21 and 32.6% PEG 6000 stress levels. The kernel correlation plot showed that the dataset correlation followed a pattern similar to that observed with the MCIA analysis, indicating that the dataset correlation was heavily influenced by stress levels (Supplementary Figure S8 A).

#### Comparison of data analysis results

The CADM test indicated that some of the obtained distance matrices were similar (CADM global test, *p* < 0.001). The most congruent were strain groupings according to the PCA based on all datasets and the PCA based on osmotic stress data (Mantel *r* = 0.54, *p* < 0.001). In addition, the MCIA and KPCA results correlated (Mantel *r* = 0.46, *p* < 0.01). The similarities among the studied data integration methods were visualized using a dendrogram based on the Mantel test correlation values (Supplementary Figure S9). The posteriori test indicated that the strain distance matrices of the KPCA and PCA based on osmotic stress data were incongruent with the rest of the distance matrices.

## Discussion

### Estimating strains difference using univariate and multivariate analysis

Among the eight strains included in this study, seven were spore-forming gram-positive *Bacillus* strains that were generally resistant to the effects of dryness, heat, UV radiation, and various other environmental stressors (Nicholson et al., 2000; Tortora et al., 2019). However, the measured biochemical and microbiological indicators revealed significant differences in osmotic stress tolerance and PGP properties among the strains.

Based on the univariate analysis results*, B*. *endophyticus* PB3, *B*. *altitudinis* PB46, and *B*. *megaterium* PB50 stood out with their phytohormone production ability, with *B*. *megaterium* PB50 producing the highest amounts of all four phytohormones. The production of these phytohormones by *B*. *megaterium* species has been reported previously (Karadeniz et al., 2006), and this ability has been associated with the stimulation of growth and stress alleviation in plants (Shahzad et al., 2017; Sun et al., 2017; Kang et al., 2019; Zerrouk et al., 2020) by a direct effect on phytohormone levels (Vejan et al., 2016).

The production of HCN and siderophores in bacterial cells serves as a defense mechanism against other microbes. While known to be good siderophore producers that inhibit the growth of several plant pathogens, *Bacillus* species have been shown to possess moderate HCN production ability (Muthukumar et al., 2022). In this study, all *Bacillus* strains could produce siderophores; however, *B*. *megaterium* PB50 was the most effective siderophore producer among the studied strains and was the only strain that produced hydroxamate-type siderophores (Ferreira et al., 2019). *Bacillus megaterium* is a dominant rice phyllosphere bacterial species that has shown antagonistic activity against several predominant rice fungal pathogens *in vitro* (Islam and Nandi, 1985; Gowdu and Balasubramanian, 1988). Moreover, *B*. *megaterium* PB50 was among the three *Bacillus* strains that showed antagonistic activity against rice fungal pathogens in this study. However, another strain, *B*. *pumilus* PB18, also exhibited antagonistic activity against fungal pathogens and had the highest siderophore production activity among the strains examined in this study.

In all strains, a decrease in IAA and GA production was observed under more pronounced osmotic stress conditions; however, the production was again the highest in *B*. *megaterium* PB50 at different stress levels. Similarly, a higher production of IAA by *B*. *megaterium* at the 15% PEG level was detected by Armada et al. (2014). In addition, decreased IAA, GA, and cytokinin production under stress conditions (20% PEG 6000) compared to non-stress conditions was observed in *Azospirillum brasilense* and *B*. *subtilis* (Ilyas et al., 2020). In addition, to *B*. *megaterium* PB50, *B*. *altitudinis* PB46 stood out from the other strains in responding to stress levels. Similar to earlier reports for different *Bacillus* species (Paul et al., 2015; Lee et al., 2018), the growth of the two aforementioned strains was faster at 11% PEG 6000 than under non-stress conditions.

Bacterial growth and proline and GB accumulation increased with increasing osmotic stress levels of up to 21% PEG 6000 in all strains. Phytohormone production decreased with increasing proline and GB concentrations. In contrast to *B*. *megaterium* PB50, which showed the lowest proline accumulation and highest phytohormone production under stress, *B*. *pumilus* PB18 showed the highest osmolyte accumulation and lowest phytohormone production under stress. Although all studied strains were gram-positive bacteria that could synthesize endogenous proline under osmotic stress conditions by boosting the synthesis process and then degrading proline for another metabolism (Tempest et al., 1970; Deutch, 2019), and the GB is transported from an external source or converted from imported choline during stress (Onyango and Alreshidi, 2018), the results of this study suggest that osmolyte accumulation is balanced. Each bacterium uses a specific osmolyte synthesis mechanism to overcome abiotic stress (Bremer and Krämer, 2019). It has been shown earlier that under non-stress conditions, bacteria favor phytohormone synthesis rather than osmolyte accumulation, whereas the opposite occurs under stress conditions. Such a shift in metabolic activity is common in bacterial cells, allowing them to maintain homeostasis when there is an increase in osmotic pressure in the extracellular medium (Varela et al., 2004; Lahtvee et al., 2014; Cesar et al., 2020).

### Integration of bacterial strain datasets

Complementary information from several types of datasets produced for microbial strains can be exploited to obtain better insights into microbial strain grouping and to elucidate factors behind strain clustering using machine-learning-based data integration methods. We applied three data-integration approaches to obtain microbial datasets: early, intermediate, and mixed integration.

In the early integration approach, several microbial datasets were combined into a single table and processed using PCA. This approach highlighted two strains, *B*. *altitudinis* PB46 and *B*. *megaterium* PB50, which were the most distinct from the other strains. At the same time, the PCA of the concatenated data did not clearly indicate the variables important for the separation of microbial strains in the PCA plot. Although the early integration approach is appealing owing to its simplicity and easy implementation, the complexity, data imbalance, and possible noise in the underlying matrix may complicate learning. As a linear dimension reduction method, PCA is the most commonly used approach for concatenated data, whereas nonlinear methods (*t*-distributed stochastic neighbor embedding, t-SNA; uniform manifold approximation, and UMAP) may provide better performance depending on the dataset properties (Xiang et al., 2021).

For mixed data integration, we utilized the MKL technique, in which a linear kernel was first computed for each microbial dataset and then the obtained kernels were combined to produce a global similarity matrix that describes microbial strains across all included datasets (Mariette and Villa-Vialaneix, 2018). The resulting similarity matrix was used as the input for the PCA. The results of this method also emphasized the separation of the two strains, *B*. *altitudinis* PB46 and *B*. *megaterium* PB50, from the other studied strains. In addition, the analysis outcome provided estimates of the variable importance. Direct PGP traits, such as GA and proline production at stress levels of 21 and 32% of PEG 6000, were highlighted by this analysis. The MKL technique has the advantage of preserving the original properties of the data and combining different data types by applying appropriate transformations (Zampieri et al., 2019). However, the MKL analysis results may be challenging to interpret because the interactions and correlations among different datasets are not always well determined.

The third method, MCIA, is an intermediate strategy of data integration, where multiple microbial datasets are jointly integrated without prior transformation. Similar to MKL, the MCIA reduces the dimensionality and complexity of datasets. The MCIA results clearly showed the distinction of strain *B*. *megaterium* PB50 from the other strains. The direct PGP trait data set (particularly GA and IAA production) and stress level 11% (GA production) were the most important indicators of the variation among the microbial strains. MCIA is considered one of the best-performing algorithms for the benchmarking of joint multi-omics dimensionality reduction approaches in the case of cancer datasets and provides an effective elucidation of relationships among the studied datasets (Cantini et al., 2021). Moreover, it can be considered a variation of the canonical correspondence analysis (CCA) method (Vahabi and Michailidis, 2022). The applicability of other CCA extensions could also be tested for simultaneous feature selection and classification in microbial datasets in the future.

The three applied data integration methods did not provide a similar grouping of the studied strains, except for the ordination of microbial strains based on the PCA of concatenated data. However, all three data integration methods indicated that the strains *B*. *altitudinis* PB46 and *B*. *megaterium* PB50 had high similarity in their biochemical properties and osmotic stress response. However, when applied together with *B*. *endophyticus* PB3 in a rice growth experiment under drought conditions, these two strains had variable effects on plant growth, biochemical properties, and gene expression (Devarajan et al., 2021). Strain *B*. *megaterium* PB50, and to a lesser extent *B*. *altitudinis* PB46, induced elevated drought tolerance in rice plants, while *B*. *endophyticus* PB3 had no effect. Simultaneously, the MCIA method placed the strain *B*. *endophyticus* PB3 close to *B*. *altitudinis* PB46 and *B*. *megaterium* PB50. Both MCIA and MKL indicate that GA production is one of the main features of microbial strains. In addition, MCIA emphasized IAA and MKL proline production at higher osmotic stress values. Several studies have reported the importance of microbial IAA, GA, and proline production in mitigating drought stress in crops (Ashry et al., 2022; Fadiji et al., 2022; Uzma et al., 2022).

One option for improving strain selection and feature identification in the future is to apply a combination of unsupervised and supervised data integration methods (Singh et al., 2019). In such cases, the outcome of plant inoculation experiments could be included in the process of data integration. It could also be that the microbial parameters included in the current data analysis did not completely cover the microbial traits required for successful plant application. In addition to biochemical properties, different omics (genome, transcriptome, proteome, and metabolome) datasets could be produced for microbial strains. The analysis of such datasets could be challenging, although there are several data integration methods specifically designed for the analysis of different omics layers.

## Conclusion

Overall, our findings suggest that when selecting bacterial strains to improve crop resilience under field drought conditions, various aspects of the PGP activity and osmotic stress tolerance of microbial strains should be considered simultaneously. Data integration methods could complement the single-table data analysis approach and may provide better insight into the microbial strain selection process. In addition, data integration allows for the exploration of complex microbial strain datasets within a single analytical framework. Currently, there are no general rules for selecting the most efficient data integration method for biological datasets. Thus, additional benchmarking of different joint data analysis methods with larger microbial strain datasets is advisable for PGP microbial strains. Another aspect of the data integration of PGP microbial strains, which needs further exploration, is related to the joint analysis of microbial biochemical and omics datasets. The integration of microbial biochemical and omics datasets may provide better insights for producing mixtures of PGP microbes that perform better than a single strain.

In this study, we used only joint data analysis to combine diverse datasets related to microbial strain properties. In the future, supervised data integration methods could be applied to combine greenhouse experiments and field trial data with PGP strain characterization data to improve the strain selection process.

## Data availability statement

The raw data supporting the conclusions of this article will be made available by the authors, without undue reservation.

## Author contributions

AD and SG designed the study. AD performed the experiments, assisted by SG and GM and wrote the first draft of the manuscript. AD and JT performed data analyses. MT, JT, SG, and GM read the first version of the manuscript. All authors contributed to the article and approved the submitted version.

## Funding

This research was funded by the Department of Biotechnology, Ministry of Science and Technology, grant number BT/IN/Indo-US/Foldscope/39/2015; Department of Science and Technology, Science and Engineering Research Board, grant number EMR/2016/008061; and Estonian Research Council (grant numbers PRG548 and PRG916).

## Conflict of interest

The authors declare that the research was conducted in the absence of any commercial or financial relationships that could be construed as a potential conflict of interest.

## Publisher’s note

All claims expressed in this article are solely those of the authors and do not necessarily represent those of their affiliated organizations, or those of the publisher, the editors and the reviewers. Any product that may be evaluated in this article, or claim that may be made by its manufacturer, is not guaranteed or endorsed by the publisher.

## Footnotes

^1^ www.ncbi.nlm.nih.gov/genbank/
