# Supplementary material for: Application of data integration for rice bacterial strain selection by combining their osmotic stress response and plant growth-promoting traits
Source: Front Microbiol. 2022 Dec 15;13:1058772. doi: 10.3389/fmicb.2022.1058772 (PMC9797599; doi:10.3389/fmicb.2022.1058772)
Supplement: Supplementary file 1 [file Data_Sheet_1.PDF]

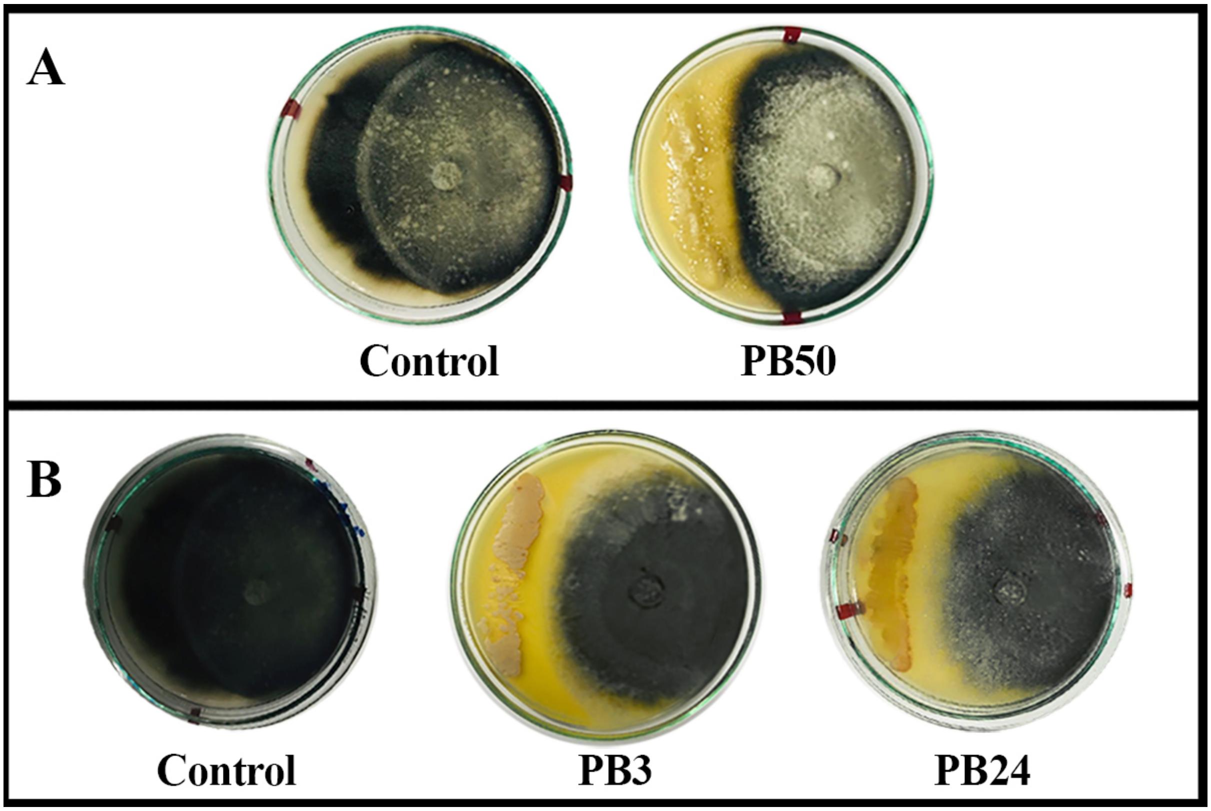

Supplementary Figure S1: Inhibition of mycelial growth of fungal pathogens by rice phyllosphere bacteria (**A**) *Helminthosporium oryzae* growth inhibition by *Bacillus megaterium* PB50 and (**B**) *Pyricularia oryzae* growth inhibition by *Bacillus endophyticus* PB3 and *Staphylococcus sciuri* PB24.

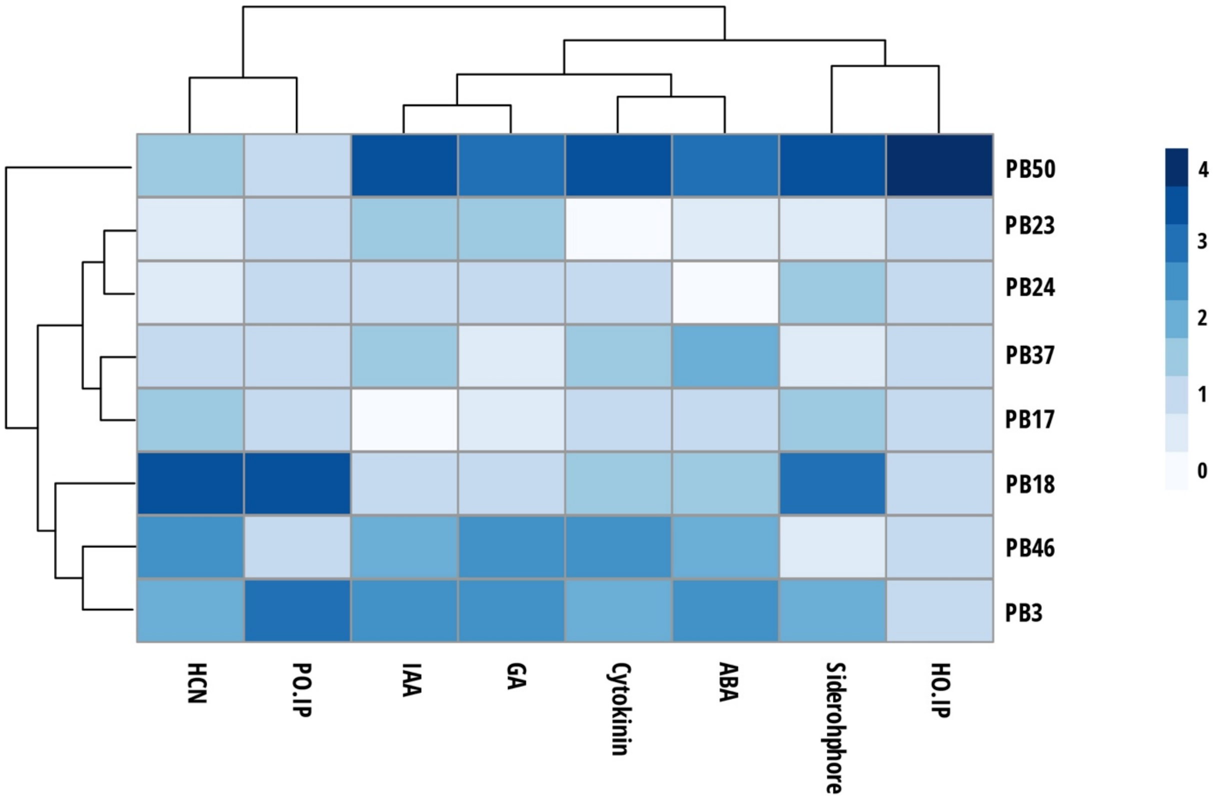

Supplementary Figure S2: Two-way heatmap showing the clustering of bacterial strains according to plant growth-promoting (PGP) traits (indole-acetic acid (IAA), gibberellic acid (GA), abscisic acid (ABA), cytokinin, siderophore, hydrogen cyanide (HCN) production, and inhibition percentage of *Helminthosporium oryzae* (HO.IP) and *Pyricularia oryzae* (PO.IP)) of rice phyllosphere bacterial strains. Strain names in the figures are *B. endophyticus* PB3, *B. australimaris* PB17, *B. pumilus* PB18, *B. safensis* PB23, *S. sciuri* PB24, *B. altitudinis* PB37, *B. altitudinis* PB46, and *B. megaterium* PB50.

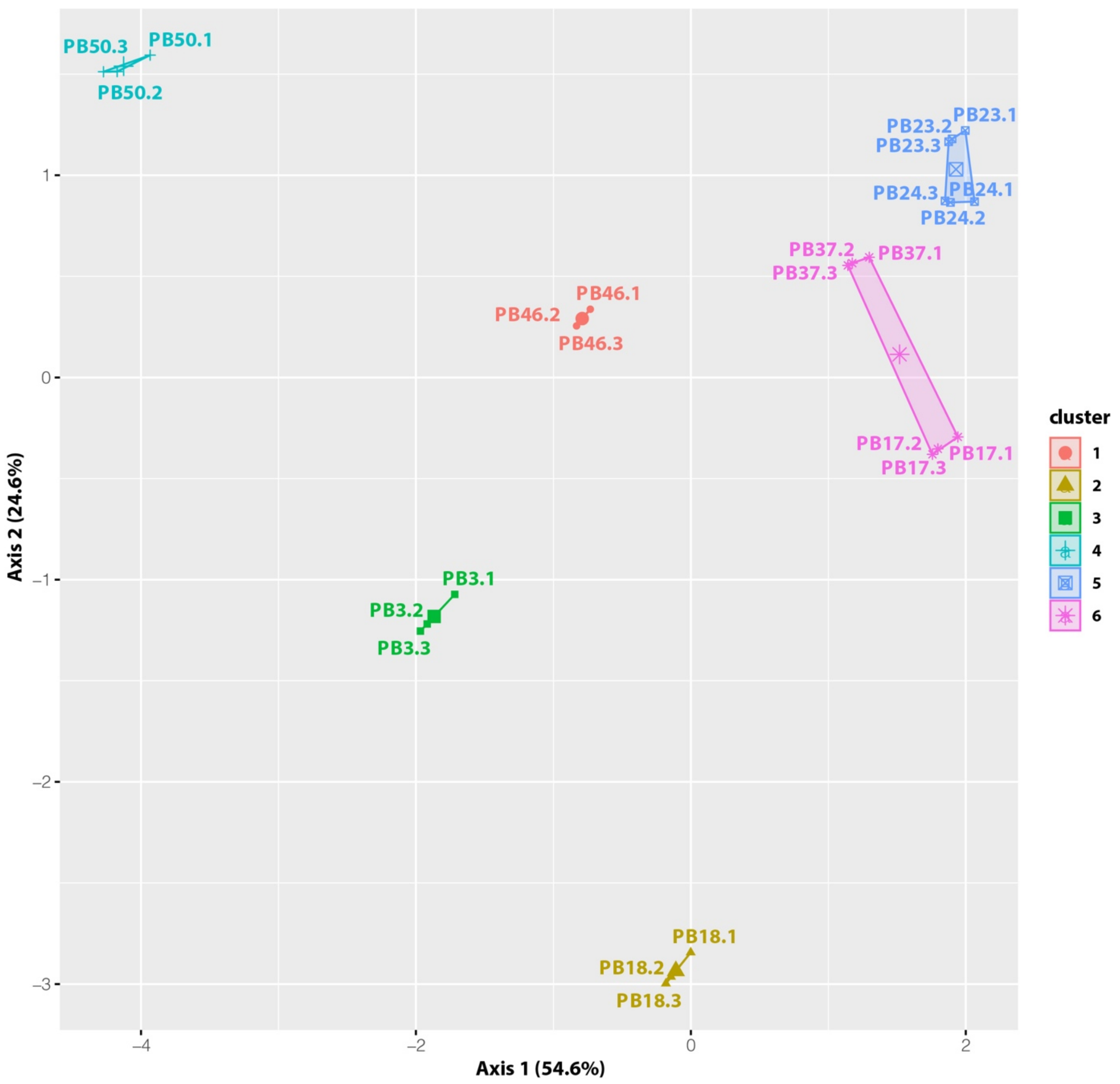

Supplementary Figure S3: Plot of k-means clustering result of bacterial strains according to plant growth-promoting (PGP) traits of bacterial strains. Optimal number of clusters was determined using silhouette score and decided as six (K = 6). Clusters are marked by colours and shapes as denoted in the cluster legends (n = 3). Strain names in the figures are *B. endophyticus* PB3, *B. australimaris* PB17, *B. pumilus* PB18, *B. safensis* PB23, *S. sciuri* PB24, *B. altitudinis* PB37, *B. altitudinis* PB46, and *B. megaterium* PB50.

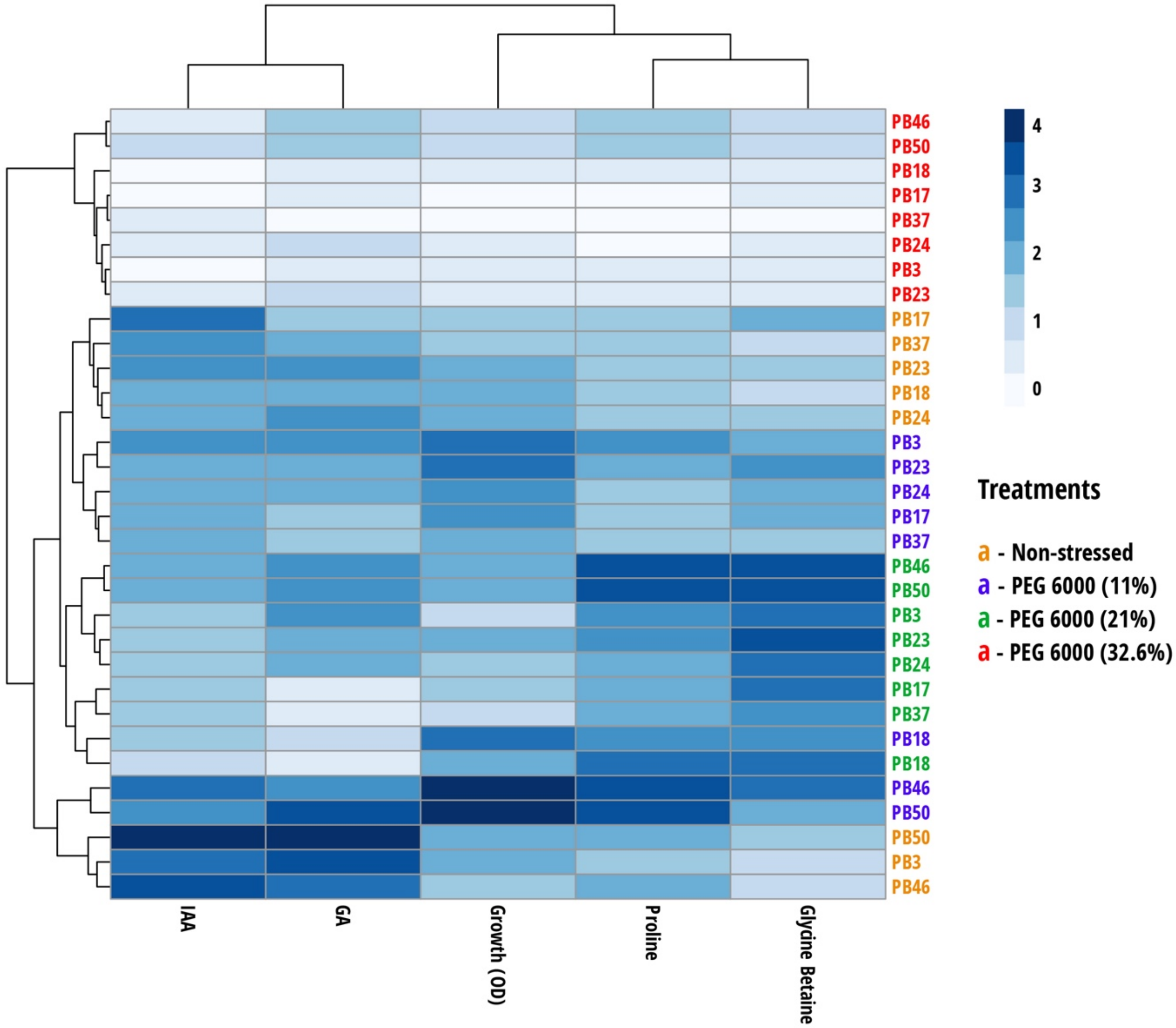

Supplementary Figure S4: Two-way heatmap showing the clustering of bacterial strains according to various microbial parameters (indole-acetic acid (IAA), gibberellic acid (GA), proline, glycine betaine production, and growth of various bacterial strains) of rice phyllosphere bacteria under three different osmotic stress conditions and in the absence of stress. Strain names in the figures are *B. endophyticus* PB3, *B. australimaris* PB17, *B. pumilus* PB18, *B. safensis* PB23, *S. sciuri* PB24, *B. altitudinis* PB37, *B. altitudinis* PB46, and *B. megaterium* PB50.

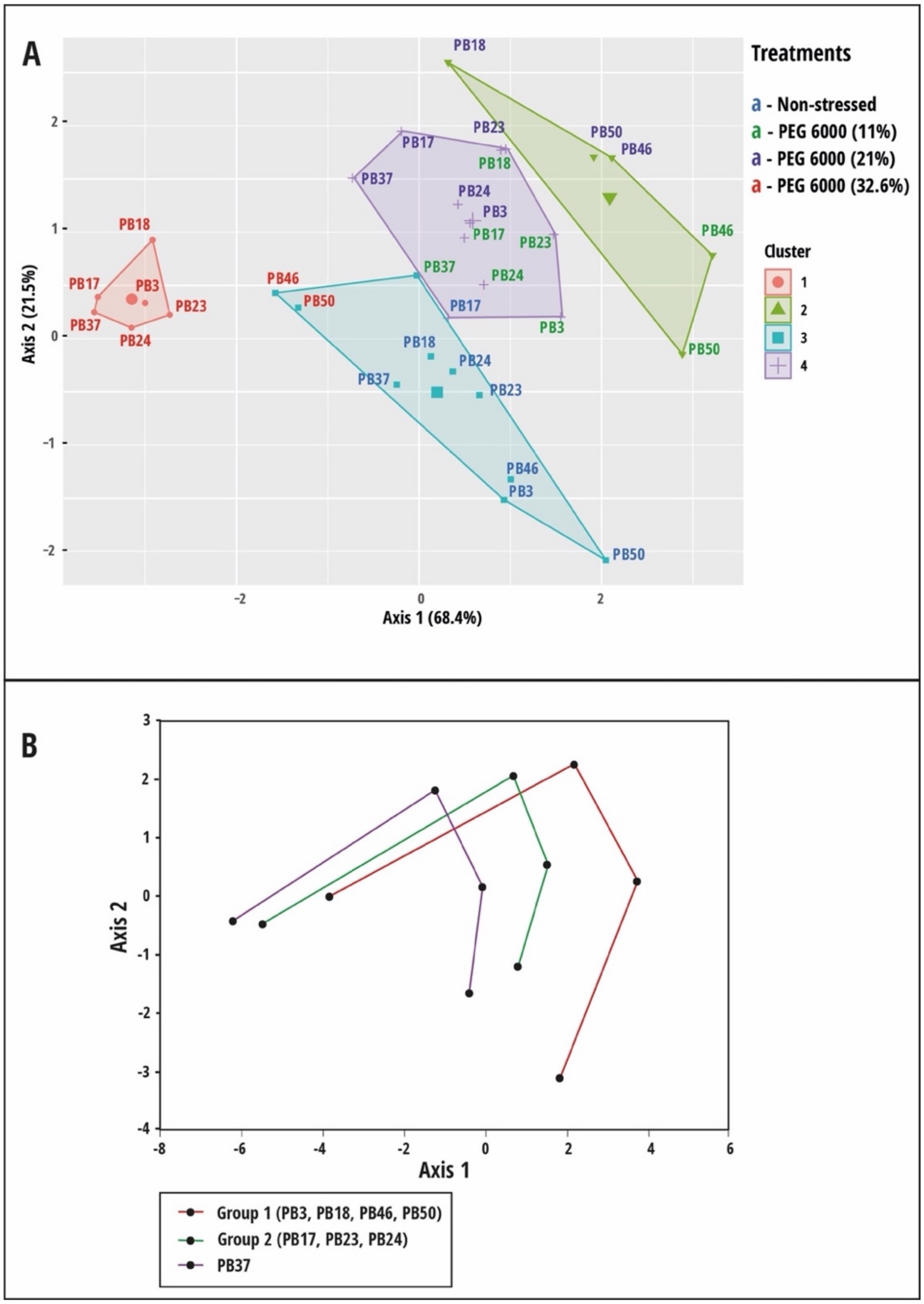

Supplementary Figure S5: (**A**) Plot of k-means clustering result of bacterial strains according to microbial parameters such as indole-acetic acid (IAA), gibberellic acid (GA), proline, glycine betaine production, and growth of various bacterial strains under different osmotic stress conditions. The optimal number of clusters was determined using silhouette score and decided as four (K = 4). Clusters are marked by colours and shapes as denoted in the cluster legends and the labels with different colours indicate the mean data of bacterial strains under different osmotic stress conditions (n = 3). (**B**) Spectral clustering plot showing the PCA sample score average values for groups of bacterial strains based on their response to different osmotic stresses. Strain names in the figures are *B. endophyticus* PB3, *B. australimaris* PB17, *B. pumilus* PB18, *B. safensis* PB23, *S. sciuri* PB24, *B. altitudinis* PB37, *B. altitudinis* PB46, and *B. megaterium* PB50.

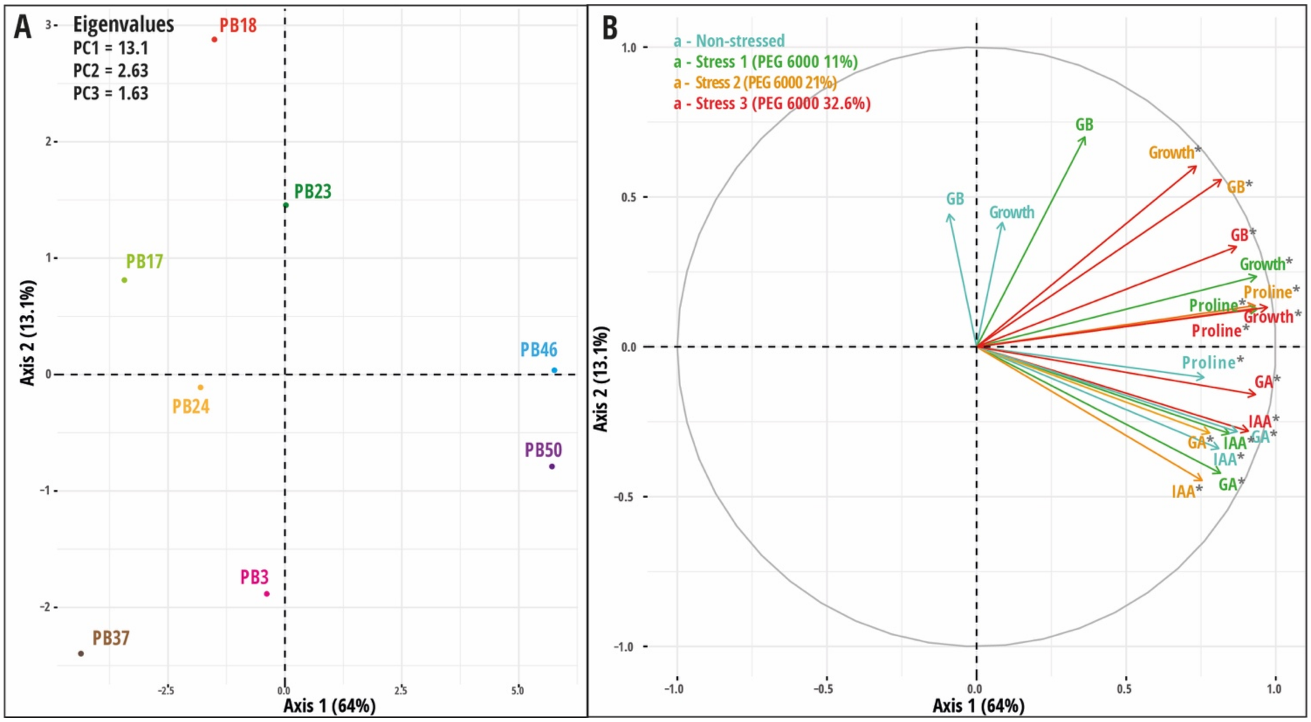

Supplementary Figure S6: Results of principal component analysis (PCA) based on the integration of osmotic stress response parameters datasets. (**A**) Score plot and (**B**) loading plot of two first principal components. The plots correspond to 64 % of the total data variance and variance proportions are shown along each principal component axis. Variables with asterisk in the plot B are significant along PC1 axis. Abbreviations used in plot (B) are, indole-acetic acid (IAA), gibberellic acid (GA), glycine betaine (GB). The codes of the strains in plot (**A**) refer to the following strains: *B. endophyticus* PB3, *B. australimaris* PB17, *B. pumilus* PB18, *B. safensis* PB23, *S. sciuri* PB24, *B. altitudinis* PB37, *B. altitudinis* PB46, and *B. megaterium* PB50.

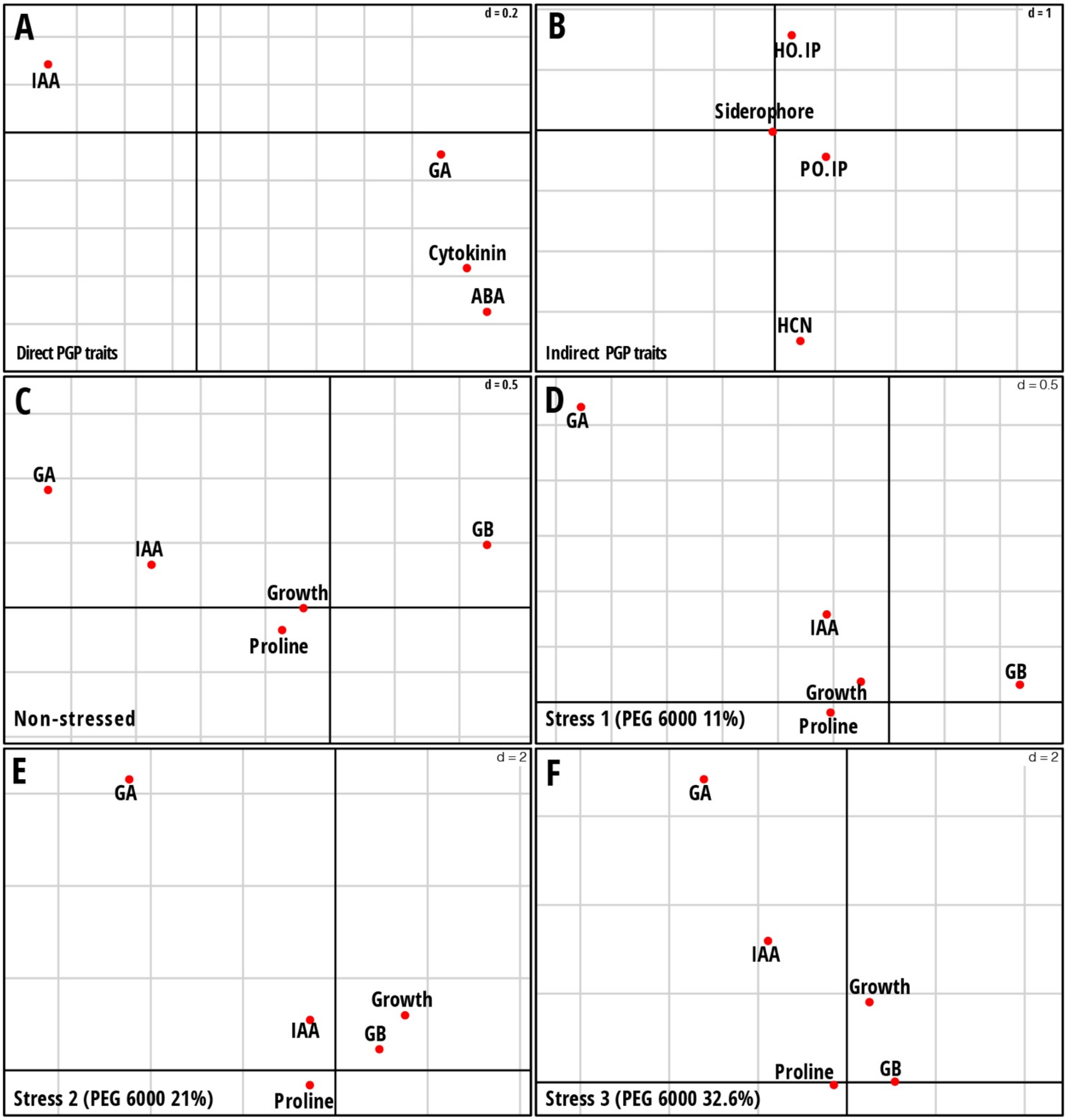

Supplementary Figure S7: Multiple co-inertia analysis results based on six data sets ((direct and indirect plant growth-promoting (PGP) traits, and microbial parameters under osmotic stress at PEG concentrations 0%, 11%, 21%, 32.6%). Shown are plots for variable spaces for each data set. Abbreviations: IAA (indole-acetic acid), GA (gibberellic acid), ABA (abscisic acid), HCN (hydrogen cyanide), HO.IP (*Helminthosporium oryzae* inhibition percentage) and PO.IP (*Pyricularia oryzae* inhibition percentage). Strain names in the figures are *B. endophyticus* PB3, *B. australimaris* PB17, *B. pumilus* PB18, *B. safensis* PB23, *S. sciuri* PB24, *B. altitudinis* PB37, *B. altitudinis* PB46, and *B. megaterium* PB50.

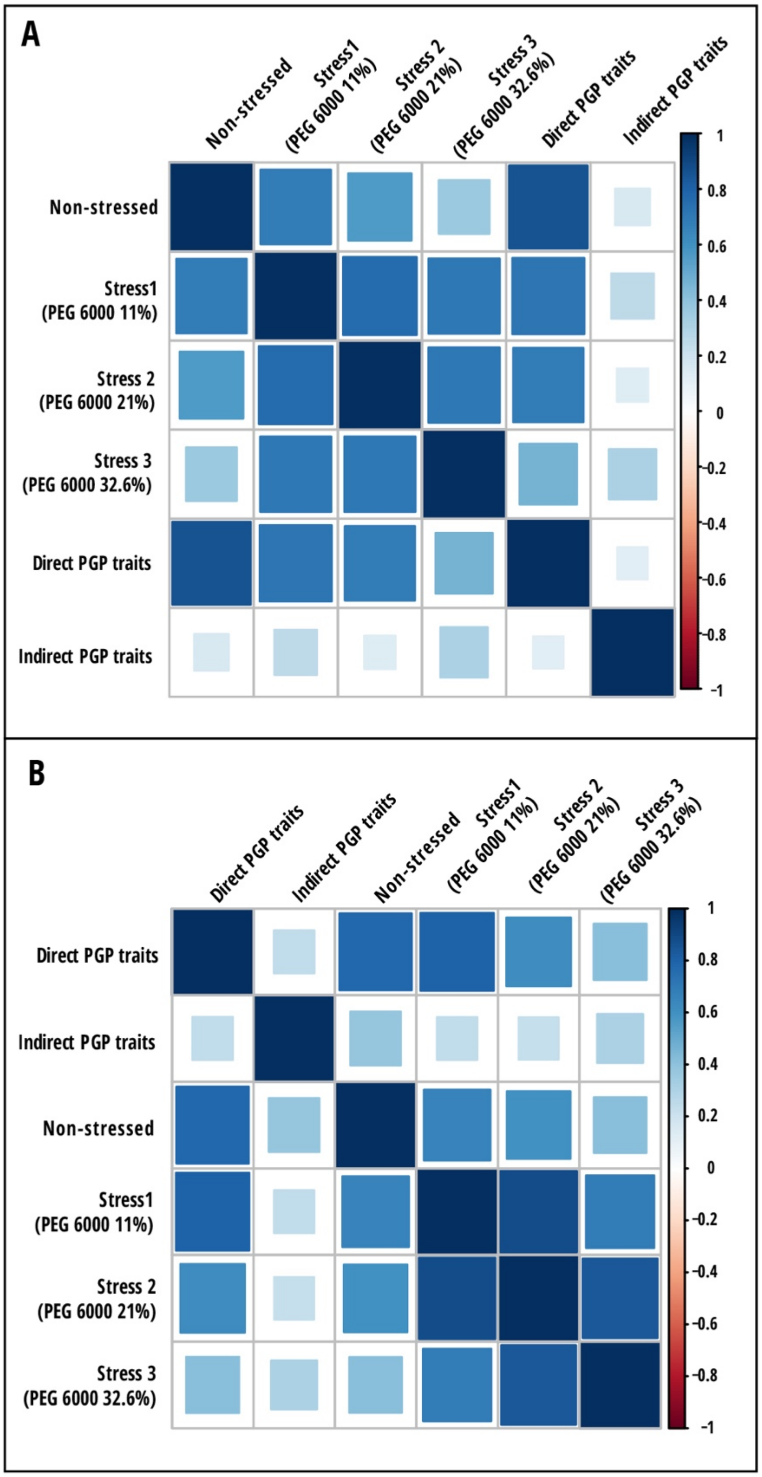

Supplementary Figure S8: Similarities between (A) multiple coinertia objects using RV coefficient values (B) kernels computed using the STATIS- UMKL approach obtained from six different datasets (direct and indirect plant growth-promoting (PGP) traits, amd microbial parameters under osmotic stress at PEG concentrations 0%, 11%, 21%, 32.6%).

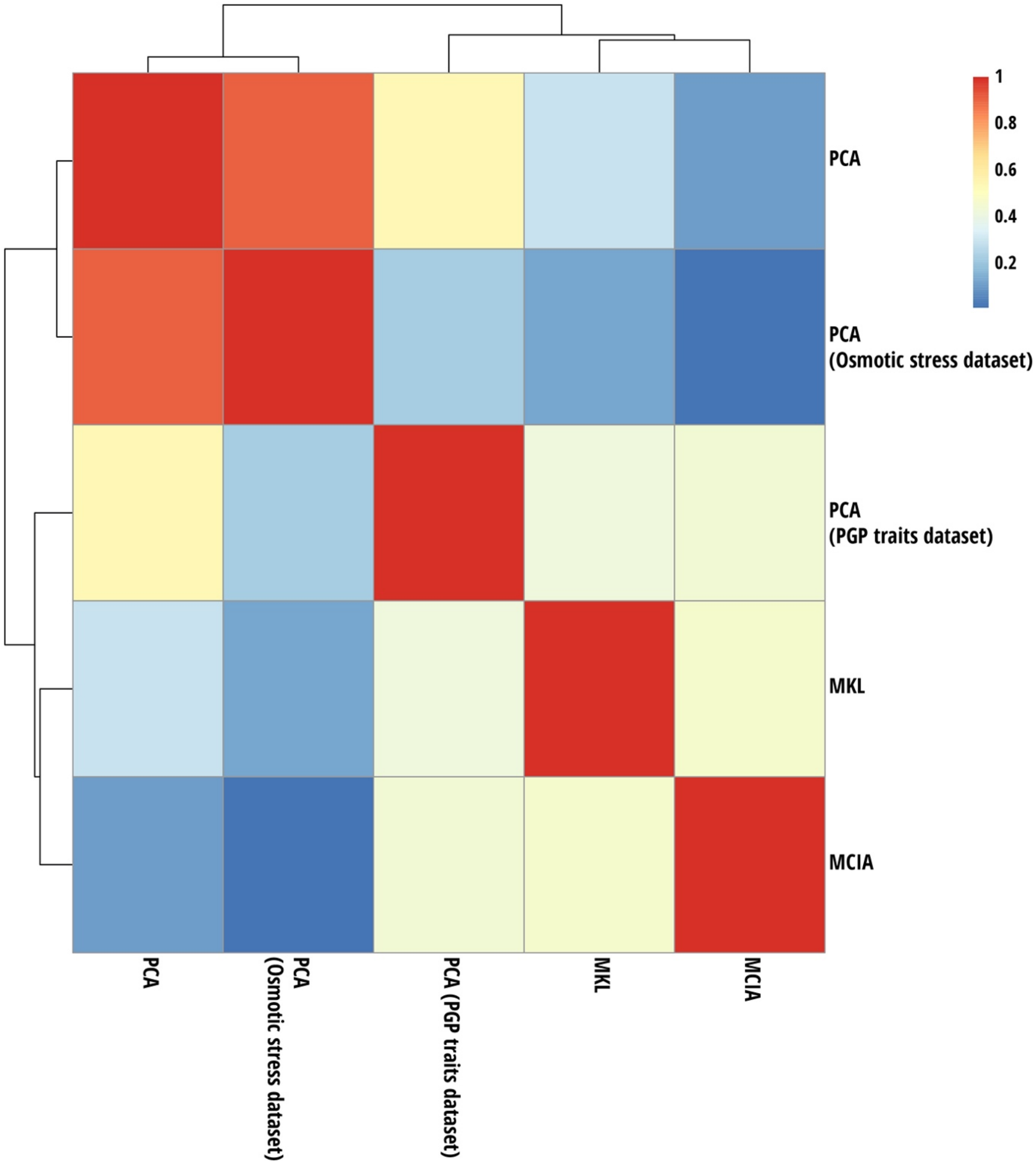

Supplementary Figure S9: Clustering of the strain ordination results based on Mantel test correlation values from Congruence among distance matrices (CADM) method.

Supplementary Table S1: List of applied data analysis methods and software.

Method	Software	Reference
One-way and two-way ANOVA	R stats	Team, R.C., 2013
One-way and two-way MANOVA	R stats	Team, R.C., 2013
Principal Component Analysis (PCA)	R package 'FactoMineR'	Lê et al., 2008
PCAtest	R package 'PCAtest'	Camargo, 2022
Multiple co-inertia analysis (MCIA)	R package 'omicade4'	Meng et al., 2013
Heatmaps	R package 'pheatmap'	Kolde and Kolde, 2018
kmeans clustering	R stats	Team, R.C., 2013
Spectral clustering	R package 'Spectrum'	John et al., 2020
KPCA	R package 'mixKernel'	Nicholson et al., 2018
Congruence among distance matrices	R package 'ape'	Paradis et al., 2019

Supplementary Table S2: One-way MANOVA results of direct and indirect plant growth-promoting traits (PGP) of different bacterial strains.

Dataset	Factors	df	Test stat	f-value	Num df	Den df	Significance
Direct PGP traits	Strains	7	3.9879	752.35	28	64	P<0.001
Indirect PGP traits	Strains	7	3.9866	680.62	28	64	P<0.001

f-value, F- statistics; Num df, number of degrees of freedom in the model; Den df, number of degrees of freedom associated with the model errors.

Supplementary Table S3: Two-way MANOVA results of different microbial parameters such as indole-acetic acid (IAA), gibberellic acid (GA), proline, glycine betaine production, and growth of various bacterial strains under different osmotic stress conditions.

Factors	df	Test stat	f-value	Num df	Den df	Significance

Strains	7	3.95	34.47	35	320	P<0.001
Osmotic stress	3	2.85	243.01	15	186	P<0.001
Strains × Osmotic stress	21	4.24	17.09	105	320	P<0.001

f-value, F- statistics; Num df, number of degrees of freedom in the model; Den df, number of degrees of freedom associated with the model errors.

Supplementary Table S4: One-way MANOVA results of different microbial parameters such as indole-acetic acid (IAA), gibberellic acid (GA), proline, glycine betaine (GB) production, and growth of various bacterial strains under different osmotic stress conditions.

**Factor 1**	**Factor 2**	**df**	**Test stat**	**f-value**	**Num df**	**Den df**	**Significance**
PEG 6000 0%	Strains	7	4.03	9.49	35	80	P<0.001
PEG 6000 11%	Strains	7	4.06	9.83	35	80	P<0.001
PEG 6000 21%	Strains	7	4.26	13.19	35	80	P<0.001
PEG 6000 32%	Strains	7	3.74	6.79	35	80	P<0.001

f-value, F- statistics; Num df, number of degrees of freedom in the model; Den df, number of degrees of freedom associated with the model errors.

Supplementary Table S5. Two-way ANOVA results of different microbial parameters such as indole-acetic acid (IAA), gibberellic acid (GA), proline, glycine betaine (GB) production, and growth of various bacterial strains under different osmotic stress conditions.

**Dependent variables**	**Factors**	**df**	**Sum of square**	**Mean square**	**f-value**	**Significance**
**IAA**	Strains	7	41.11	5.87	1917.6	P<0.001
	Osmotic stress	3	234.52	78.17	25528.1	P<0.001
	Strains × Osmotic stress	21	12.76	0.61	198.4	P<0.001
	Residuals	64	0.20	0.00		
**GA**	Strains	7	181.71	25.96	989.95	P<0.001

	Osmotic stress	3	166.97	55.66	2122.47	P<0.001
	Strains × Osmotic stress	21	22.13	1.05	40.18	P<0.001
	Residuals	64	1.68	0.03		
**Proline**	Strains	7	480891	68699	955.95	P<0.001
	Osmotic stress	3	1354206	451402	6281.32	P<0.001
	Strains × Osmotic stress	21	108249	5155	71.73	P<0.001
	Residuals	64	4599	72		P<0.001
**Glycine betaine**	Strains	7	79045	11292	823.7	P<0.001
	Osmotic stress	3	1118652	372884	27198.8	P<0.001
	Strains × Osmotic stress	21	72240	3440	250.9	
	Residuals	64	877	14		
**Growth (OD)**	Strains	7	0.619	0.0885	14.870	P<0.001
	Osmotic stress	3	4.452	1.4841	249.484	P<0.001
	Strains × Osmotic stress	21	0.390	0.0186	3.125	P<0.001
	Residuals	64	0.381	0.0059		

Factor, independent variables (strains, osmotic stress), and their interaction (strains × osmotic stress). Abbreviations. df, degrees of freedom; F, the value for comparison with the critical value for significance; p, the level of significance.

Supplementary Table S6. One-way ANOVA results of different microbial parameters such as indole-acetic acid (IAA), gibberellic acid (GA), proline, glycine betaine (GB) production, and growth of various bacterial strains at PEG 6000 stress level 0%.

**Dependent variables**	**Factor**	**df**	**Sum square**	**Mean square**	**f-value**	**Significance**
IAA	Strains	7	31.84	4.549	1631	P<0.001
	Residuals	16	0.04	0.003		
GA	Strains	7	59.01	8.430	112.7	P<0.001
	Residuals	16	1.2	0.075		
Proline	Strains	7	6444	920.6	38.29	P<0.001
	Residuals	16	385	24		

GB	Strains	7	44375	6339	346	P<0.001
	Residuals	16	293	18		
Growth	Strains	7	0.1025	0.01465	1.878	NS
	Residuals	16	0.1248	0.00780		

df, degrees of freedom; F, the value for comparison with the critical value for significance; p, the level of significance. NS, not significant.

Supplementary Table S7. One-way ANOVA results of different microbial parameters such as indole-acetic acid (IAA), gibberellic acid (GA), proline, glycine betaine (GB) production, and growth of various bacterial strains at PEG 6000 stress level 11%.

**Dependent variables**	**Factor**	**df**	**Sum square**	**Mean square**	**f-value**	**Significance**
IAA	Strains	7	11.598	1.657	843.4	P<0.001
	Residuals	16	0.31	0.002		
GA	Strains	7	57.50	8.214	876.5	P<0.001
	Residuals	16	0.15	0.009		
Proline	Strains	7	195974	27996	1363	P<0.001
	Residuals	16	329	21		
GB	Strains	7	44960	6423	472.8	P<0.001
	Residuals	16	217	14		
Growth	Strains	7	0.526	0.0751	7.059	P<0.001
	Residuals	16	0.1703	0.01065		

df, degrees of freedom; F, the value for comparison with the critical value for significance; p, the level of significance. NS, not significant.

Supplementary Table S8. One-way ANOVA results of different microbial parameters such as indole-acetic acid (IAA), gibberellic acid (GA), proline, glycine betaine (GB) production, and growth of various bacterial strains at PEG 6000 stress level 21%.

**Dependent variables**	**Factor**	**df**	**Sum of square**	**Mean square**	**f-value**	**Significance**
IAA	Strains	7	5.730	0.8186	243.1	P<0.001
	Residuals	16	0.054	0.0034		

GA	Strains	7	50.58	7.225	774	P<0.001
	Residuals	16	0.15	0.009		
Proline	Strains	7	188181	26883	3219	P<0.001
	Residuals	16	134	8		
GB	Strains	7	46546	6649	321	P<0.001
	Residuals	16	331	21		
Growth	Strains	7	0.26276	0.03754	32.3	P<0.001
	Residuals	16	0.0185	0.00116		

df, degrees of freedom; F, the value for comparison with the critical value for significance; p, the level of significance. NS, not significant.

Supplementary Table S9. One-way ANOVA results of different microbial parameters such as indole-acetic acid (IAA), gibberellic acid (GA), proline, glycine betaine (GB) production, and growth of various bacterial strains at PEG 6000 stress level 32.6%.

**Dependent variables**	**Factor**	**df**	**Sum of square**	**Mean square**	**f-value**	**Significance**
IAA	Strains	7	4.694	0.6706	162.4	P<0.001
	Residuals	16	0.066	0.0041		
GA	Strains	7	18.166	2.5952	578.1	P<0.001
	Residuals	16	0.072	0.0045		
Proline	Strains	7	199011	28430	135.8	P<0.001
	Residuals	16	3349	209		
GB	Strains	7	15406	2200.8	991.1	P<0.001
	Residuals	16	36	2.2		
Growth	Strains	7	0.1182	0.016888	4.033	P<0.001
	Residuals	16	0.0670	0.004187		

df, degrees of freedom; F, the value for comparison with the critical value for significance; p, the level of significance. NS, not significant.

Supplementary Table S10. Dataset combination type used for integration methods

**Techniques**	**Datasets**	**Variables**
**PCA, MKL & MCIA**	Dataset 1: Direct PGP traits	IAA, GA, ABA and cytokinin
	Dataset 2: Indirect PGP traits	Siderophore, HCN, Inhibition percentage of *Pyricularia oryzae* and *Helminthosporium oryzae*
	Dataset 3: Non-stressed	IAA, GA, GB, Proline and growth
	Dataset 4: Stress 1 (PEG 6000 11%)	IAA, GA, GB, Proline and growth
	Dataset 5: Stress 2 (PEG 6000 21%)	IAA, GA, GB, Proline and growth
	Dataset 6: Stress 3 (PEG 6000 32.6%)	IAA, GA, GB, Proline and growth

Abbreviations used are PGP (plant growth promotion), IAA (indole-acetic acid), GA (gibberellic acid), ABA (abscisic acid), HCN (hydrogen cyanide) and GB (glycine betaine).

Supplementary Table S11. The results of significant variable loading on the principal component axes (PC1 and PC2), as well as variable loading values from the PGP traits dataset, the osmotic stress experiment dataset, and their combination dataset.

**PGP traits dataset**			**Osmotic stress experiment dataset**			**PGP traits + Osmotic stress experiment dataset**		
**Eigenvalues:** **(PC1 = 4.36; PC2 = 1.96; PC3 = 0.86)**			**Eigenvalues:** **(PC1 = 10.1; PC2 = 3.27; PC3 = 0.83)**			**Eigenvalues:** **(PC1 = 16.0; PC2 = 3.63; PC3 = 3.39)**		
**Variables**	**PC axis**	**Loading values**	**Variables**	**PC axis**	**Loading values**	**Variables**	**PC axis**	**Loading values**
Siderophore	PC1	2.49	IAA	PC1	2.64	IAA (Non-stressed)	PC1	12.6
HCN	PC2	2.07	GA	PC1	2.42	GA (Non-stressed)	PC1	12.5
PO.IP	PC2	1.81	Proline	PC1	2.96	Proline (Non-stressed)	PC1	9.36
HO.IP	PC1	3.07	GB	PC1	2.14	GB (Non-stressed)	PC2	2.58
IAA	PC1	3.57	Growth	PC1	2.78	Growth (Non-stressed)	PC2	2.54
GA	PC1	3.31				IAA (Stress 1 - PEG 6000 11%)	PC1	13.1
Cytokinin	PC1	3.84				GA (Stress 1 - PEG 6000 11%)	PC1	12.2
ABA	PC1	3.55				Proline (Stress 1 - PEG 6000 11%)	PC1	14.1
						GB (Stress 1 - PEG 6000 11%)	PC2	3.83
						Growth (Stress 1 - PEG 6000 11%)	PC1	13.2
						IAA (Stress 2 - PEG 6000 21%)	PC1	9.71
						GA (Stress 2 - PEG 6000 21%)	PC1	10.4
						Proline (Stress 2 - PEG 6000 21%)	PC1	13.9
						GB (Stress 2 - PEG 6000 21%)	PC1	10.2

						Growth (Stress 2 - PEG 6000 21%)	PC1	8.49
						IAA (Stress 3 - PEG 6000 32.6%)	PC1	12.6
						GA (Stress 3 - PEG 6000 32.6%)	PC1	13.7
						Proline (Stress 3 - PEG 6000 32.6%)	PC1	14.1
						GB (Stress 3 - PEG 6000 32.6%)	PC1	12.2
						Growth (Stress 3 - PEG 6000 32.6%)	PC1	14.3
						Siderophore	PC2	4.17
						HCN	PC2	3.44
						PO.IP	PC2	3.22
						HO.IP	PC1	8.63
						IAA	PC1	11.4
						GA	PC1	13.8
						Cytokinin	PC1	10.7
						ABA	PC1	8.23

Abbreviations used are PGP (plant growth promotion), IAA (indole-acetic acid), GA (gibberellic acid), ABA (abscisic acid), HCN (hydrogen cyanide), HO.IP (*Helminthosporium oryzae* inhibition percentage) and PO.IP (*Pyricularia oryzae* inhibition percentage) and GB (glycine betaine).
